# Muscle Oximetry in Sports Science: An Updated Systematic Review

**DOI:** 10.1007/s40279-023-01987-x

**Published:** 2024-02-12

**Authors:** Stephane Perrey, Valentina Quaresima, Marco Ferrari

**Affiliations:** 1 EuroMov Digital Health in Motion, University of Montpellier, IMT Mines Ales, Montpellier, France; 2 Department of Life, Health and Environmental Sciences, University of L’Aquila, L’Aquila, Italy

## Abstract

**Background:**

In the last 5 years since our last systematic review, a significant number of articles have been published on the technical aspects of muscle near-infrared spectroscopy (NIRS), the interpretation of the signals and the benefits of using the NIRS technique to measure the physiological status of muscles and to determine the workload of working muscles.

**Objectives:**

Considering the consistent number of studies on the application of muscle oximetry in sports science published over the last 5 years, the objectives of this updated systematic review were to highlight the applications of muscle oximetry in the assessment of skeletal muscle oxidative performance in sports activities and to emphasize how this technology has been applied to exercise and training over the last 5 years. In addition, some recent instrumental developments will be briefly summarized.

**Methods:**

Preferred Reporting Items for Systematic Reviews guidelines were followed in a systematic fashion to search, appraise and synthesize existing literature on this topic. Electronic databases such as Scopus, MEDLINE/PubMed and SPORTDiscus were searched from March 2017 up to March 2023. Potential inclusions were screened against eligibility criteria relating to recreationally trained to elite athletes, with or without training programmes, who must have assessed physiological variables monitored by commercial oximeters or NIRS instrumentation.

**Results:**

Of the identified records, 191 studies regrouping 3435 participants, met the eligibility criteria. This systematic review highlighted a number of key findings in 37 domains of sport activities. Overall, NIRS information can be used as a meaningful marker of skeletal muscle oxidative capacity and can become one of the primary monitoring tools in practice in conjunction with, or in comparison with, heart rate or mechanical power indices in diverse exercise contexts and across different types of training and interventions.

**Conclusions:**

Although the feasibility and success of the use of muscle oximetry in sports science is well documented, there is still a need for further instrumental development to overcome current instrumental limitations. Longitudinal studies are urgently needed to strengthen the benefits of using muscle oximetry in sports science.

**Supplementary Information:**

The online version contains supplementary material available at 10.1007/s40279-023-01987-x.

## Key Points

**Table:** 

Near-infrared spectroscopy (NIRS), on which commercially available oximeters are based, is a useful complementary method for non-invasively assessing, with good sensitivity, skeletal muscle oxygen delivery and utilization in response to different exercise modes, training interventions and ergogenic aids.
NIRS instrumentation can assess a large panel of muscles at single measurement sites.
NIRS is a functional tool in which skeletal muscle oxygenation data can be viewed in ‘real time’, complementing external power and heart rate data, allowing coaches and physical trainers to make better-informed decisions to guide training or recovery processes.
Multi-modal techniques and sophisticated multi-channel NIRS instrumentations are required to provide a more detailed evaluation of muscle activity data during active exercise for a better understanding of muscle function.

## Introduction

Muscle oximetry, based on near-infrared spectroscopy (NIRS), is able to provide, non-invasively, information about the changes in oxygenation of haemoglobin (Hb), present primarily in smal vessels (< 1 mm in diameter) such as the capillary, arteriolar and venular bed, and myoglobin (Mb), a cytoplasmic protein of the striated muscles [1–3]. Considering that Mb oxygenation is expected to remain almost unchanged during exercise, any alteration of the [Hb + Mb] signal reflects mainly changes in Hb. NIRS has been implemented in three main modalities that differ from each other based on the temporal characteristics of the employed light: continuous-wave NIRS (CW-NIRS: light with constant intensity), frequency-domain NIRS (FD-NIRS: modulated light intensity) and time-domain NIRS (TD-NIRS: pulsed light intensity). CW-NIRS, based on constant tissue illumination, measures only the light attenuation through the muscle. FD-NIRS, which illuminates the muscle with intensity-modulated light, measures both the attenuation and the phase delay of the emerging light. TD-NIRS, by illuminating the muscle with short pulses of light, detects the shape of the pulse after propagation through the tissue. The quantification of muscle oxygenation depends on the NIRS technology adopted. Since the nineties, oximeters (utilizing spatially resolved CW-NIRS) have been made available for monitoring brain and muscle oxygen (O_2_) saturation (SmO_2_). The market launch of commercial relatively low-cost portable wireless muscle oximeters dates back to 2006, and our 2018 systematic review [4] highlighted the application of muscle oximetry in the assessment of skeletal muscle oxidative performance in sports activities. Fifty-seven studies published over 11 years were included in Ref. [4], emphasizing the application of this technology to physical exercise and training.

Afterwards, in 2019, Barstow [1] summarized the most common methodologies of skeletal muscle NIRS, their strengths and limitations, and discussed some of the potential confounding factors that may affect the quality and reproducibility of NIRS data in skeletal muscle. Recommendations to reduce variability and errors in data collection, analysis and interpretation were also provided. Salvatore et al. [5] summarized the effects of aging in healthy individuals and muscle O_2_ utilization; aging reduces SmO_2_ at rest, and during submaximal and maximal exercise, and extends the timeframe for restoration of SmO_2_ following exercise. In 2021, Cornelis et al. [6], summarizing the results of 11 clinical trials on the impact of exercise therapy on lower limb SmO_2_ evaluated by NIRS in patients with lower-extremity artery disease, showed that exercise training improved the de-oxygenation and re-oxygenation patterns. Recently, Tuesta et al. [7] reviewed 18 clinical trials that evaluated the effects of physical exercise on SmO_2_ in subjects with different pathologies. Muscle oximetry made it possible to observe changes in muscle oxygenation/deoxygenation parameters such as SmO_2_, oxyhaemoglobin (O_2_Hb), total haemoglobin (tHb) and deoxyhaemoglobin (HHb) upon exercise interventions in patients with chronic diseases and in healthy active subjects. NIRS is currently considered to be a particularly promising wearable biosensor. This technology enables the continuous monitoring of physiological signals at the muscle site, thereby facilitating more accurate diagnoses and follow-up examinations pertaining to local exercise metabolism and adaptation in skeletal muscle performance.

In the last 5 years, several articles have been published focusing on the technical aspects of muscle NIRS, the interpretation of the signals and the benefits of utilizing NIRS technique to measure the physiological status of muscles and to determine how the working muscles are being used [1, 3, 8, 9]. Within the field of sports science field and beyond, NIRS monitoring has a huge potential that is often ignored in an applied exercise environment [9].

Considering the consistent number of studies on the application of muscle oximetry in sports science published in the last 5 years, the objectives of this updated systematic review were to highlight the most recent applications of muscle oximetry in the assessment of skeletal muscle oxidative performance in different sports activities and to emphasize how this technology has been applied to exercise and training involving different interventions in sporting environments. In addition, some recent instrumental developments are briefly summarized.

## Methods

### Literature Search Methodology

A thorough systematic search of the research literature was performed conforming to the Preferred Reporting Items for Systematic Reviews (PRISMA) statement [10]. Our search of the literature began in December 2022 and continued through to March 2023. A search of electronic databases was conducted to identify all publications which utilized muscle oximetry in sport science published from March 2017 up to March 2023. As a prerequisite, all studies should have been performed in healthy sports populations including both adolescents and adults. Three databases (Scopus, MEDLINE/PubMed, and SPORTDiscus via the EBSCOHost) were searched electronically from inception using the terms ‘‘near infrared spectroscopy’’ OR “NIRS”, OR “oximetry” OR ‘‘muscle oxygenation”, AND with the term ‘‘sports”. Additionally, these four terms were combined (AND) with terms of different sports (“athletics” OR “badminton” OR “baseball” OR “basketball” OR “biathlon” OR “bicycling” OR “boxing” OR “canoeing” OR “climbing” OR “cricket” OR “croquet” OR “cross country” OR “cycling” OR “decathlon” OR “diving” OR “field hockey” OR “football” OR “futsal” OR “golf” OR “gymnastics” OR “handball” OR “high jump” OR “hockey” OR “ice hockey” OR “ice skating” OR “inline skates” OR “judo” OR “jumping” OR “karate” OR “kayaking” OR “kickball” OR “lacrosse” OR “long jump” OR “martial arts” OR “Nordic skiing” OR “paddling” OR “pentathlon” OR “ping pong” OR “polo” OR “racquetball” OR “rafting” OR “rock climbing” OR “roller skating” OR “rowing” OR “rugby” OR “running” OR “sailing” OR “skiing” OR”sledding” OR “snowboarding” OR “soccer” OR “softball” OR “speed skating” OR “squash” OR “Sumo wrestling” OR “surfing” OR “swimming” OR “synchronized swimming” OR “table tennis” OR “taekwondo” OR “tennis” OR “triathlon” OR “triple jump” OR “ultramarathon” OR “volley ball” OR “water polo” OR “waterskiing” OR”weightlifting” OR “white water rafting” OR “windsurfing”. Each database automatically uses its own term mapping. The results were screened to identify relevant studies, first by title, then by abstract, and finally by full text. Non-relevant titles and abstracts were omitted. full texts were screened for inclusion criteria and were only included in the review if they met all criteria. Differences in search outcomes were verified and consensus for inclusion was reached. We also extended the search scope to include “related articles”. Reference lists of fully evaluated publications were also examined for studies not found in the online database searches. Authors of published papers were also contacted directly if crucial data were not reported in the original paper.

### Inclusion and Exclusion Criteria

The following inclusion criteria were used to select articles to be included in the systematic review:

1. Only full articles published in English in peer-reviewed journals were considered. Book chapters and Proceedings were not included.
2. Studies performed in healthy sports populations including both adolescents (over 15 years) and adults were included.
3. Only studies performed using commercial oximeters or NIRS instruments that measure SmO_2_, and/or O_2_Hb and HHb changes utilizing the modified Beer–Lambert law were included.
4. Muscle studies in which physiological variables were monitored in recreationally trained to elite athletes with or without training programme were included.
5. NIRS muscle studies that did not report the oxygenation data units were discarded.

### Study Selection and Data Extraction

Regarding the study eligibility, titles and abstracts of potentially relevant articles were screened independently by two reviewers (M.F. and V.Q.). The explicit rule was to select studies that could possibly meet the inclusion criteria. Title duplicate publications were removed, and articles which did not meet the inclusion criteria were excluded. Full texts were assessed for eligibility by the three authors, and any articles that were ambiguous regarding inclusion were independently assessed against the eligibility criteria. Disagreements regarding inclusion of ambiguous articles were discussed and a consensus was agreed. A pre-designed data extraction form was used to collate data from individual studies, including country/setting, study design, characteristics of participants, representativeness of the study sample and results. Data for each included study were extracted by two reviewers (M.F. and V.Q.) and were checked by a third reviewer (S.P.). For each article, a standardized document form was used to extract the following relevant information from the selected papers: the type of sport(s), authors, publication data, sample size, participant characteristics (age, sex, body mass, training status), exercise protocol, NIRS instrumentation and related-measured variables, outcome measures with training intervention if any, muscle(s) assessed, and a summary of main findings. All these data are included in the Table S1 of the Electronic Supplementary Material [ESM] available in the online version; the Table S1 includes 191 studies [11–201]. We decided to not assess risk of bias due to several limitations of existing tools for assessing risk of reporting biases in systematic reviews [202].

## Results

**Fig. 1:**
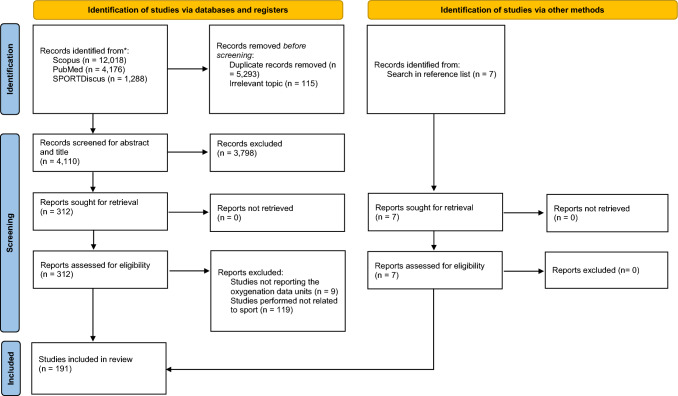
Preferred Reporting Items for Systematic reviews and Meta-Analyses (PRISMA) flow diagram of search strategy PRISMA illustrating the systematic review process and the inclusion and exclusion of research papers

Figure 1 shows the flow of information through the systematic reviewing process. Of the total 12,018 (Scopus), 4176 (PubMed) and 1288 (SPORTDiscus) articles retrieved, 5408 were excluded as title duplicate publications (5293) and irrelevant topic (115). Other 7964 records were also excluded from the remaining 12,074 because they were not pertinent. A further 3798 records were excluded from the remaining 4110 after screening of the titles and the content of the abstracts; the remaining articles were 312. Nine studies that did not report oxygenation data units and 119 studies did not relate to sport were excluded from the 312 articles; the remaining articles were 184. Further 7 articles were identified from the reference list of other articles and were added to the 184 articles. Therefore, 191 studies were retained for inclusion in the final stage of this review. Study characteristics are summarized in Table S1.

### Sporting Disciplines and Characteristics

For the purposes of reporting and analysis, the 191 included studies with 3435 participants were grouped into 37 sport disciplines (Table 1). The included studies examined recreational to elite athletes from a broad range of individual sports (e.g. cycling, rowing, skiing) and team-based sports (e.g. basketball, football, rugby). Of note, a total of eight studies included more than three sports [185–192] and a total of nine studies did not report the sporting discipline in their design [193–201]. The most common sports activities used in the 191 studies are cycling (*n* = 55), running (*n* = 23), and climbing (*n* = 11). Of the 191 studies, the majority utilized male participants (*n* = 119), 64 studies included males and females, and 8 studies included females only. Twenty-one studies reported a training intervention. A total of 53 studies (Table 1) included elite or highly trained participants, while 29 studies included well-trained participants, 51 studies included trained participants, and moderately trained participants were included in 58 studies. Of note, 16 studies were targeted especially on elite athletes, 3 studies compared young and old participants [66, 195, 197] and 7 studies investigated junior athletes (≤ 18 years) [15, 18, 60, 68, 70, 101, 161].

**Table 1:** Overview of some characteristics (in terms of numbers *N*) for the different sports included in the review

	Sport	Studies	Participants	Gender			Training level state				Wearable device	Assessed muscles
				F	M	F/M	E	WT	T	MT		
1	Alpine skiing	1	17			1	1				1	1
2	American football	1	16		1			1			1	1
3	Badminton	2	16		1	1	2				2	1
4	Basketball	5	81		4	1	4	1			4	2
5	Climbing	11	279		5	6	1	6	3	1	9	4
6	Combat sport (jiu-jitsu, judo)	4	51		3	1	2			2	3	3
7	Cross-country skiing	3	39		2	1	1	2			3	5
8	Cycling	55	907		34	21	10	10	15	20	33	7
9	Diving	3	43		3			2	1		3	2
10	Football	9	158	4	5		6		1	2	7	5
11	Futsal	1	13	1						1	1	1
12	Kayak	3	23		2	1	2		1		2	5
13	Lacrosse	1	7		1					1		1
14	Long-track speed skating	4	36		1	3	3	1			4	1
15	Marathon	4	75		3	1	1		1	2	4	4
16	Nordic walking	1	30			1				1	1	1
17	Resistance training	5	61		3	2			5		4	4
18	Roller ski skating	1	9		1		1				1	2
19	Rowing	3	35		3		1		2		3	1
20	Rugby	1	22		1		1				1	1
21	Rugby 7	2	28		2		2				2	1
22	Running	23	623		16	7	6	2	7	8	15	4
23	Speed skating	1	27			1	1				1	1
24	Sprint (track field)	1	9		1				1			
25	Sprint canoe-kayak	3	51		1	2	1	1	1		2	3
26	Swimming	5	73		2	3	2		3		4	4
27	Team sports	6	113	1	4	1	1		1	4	4	2
28	Triathlon	5	70		3	2	1		4		5	2
29	Ultra running	3	52		1	2	1	1	1		1	2
30	Cross-country running and swimming/rowing	1	19		1			1				4
31	Cycling and running	1	10			1				1	1	1
32	Cycling and triathlon	1	13		1			1			1	1
33	Handball and Football	1	9		1				1			
34	Judo and cycling	1	30		1		1				1	1
35	Running and triathlon	1	11		1		1				1	1
36	Cross-country, CrossFit, or power lifting	1	24		1				1			1
37	More than three	8	133	1	5	2			2	6	2	5
38	Not reported	9	222	1	5	3				9	3	7
Total		191	3435	8	119	64	53	29	51	58	130	–

*E* elite or highly trained participants,* F* female,* F/M* female and male,* M* male,* MT* moderately trained participants, *N* number of studies,* T* trained participants,* WT* well-trained participants. Note that wearable devices were Humon Hex, Moxy or Portamon

**Fig. 2:**
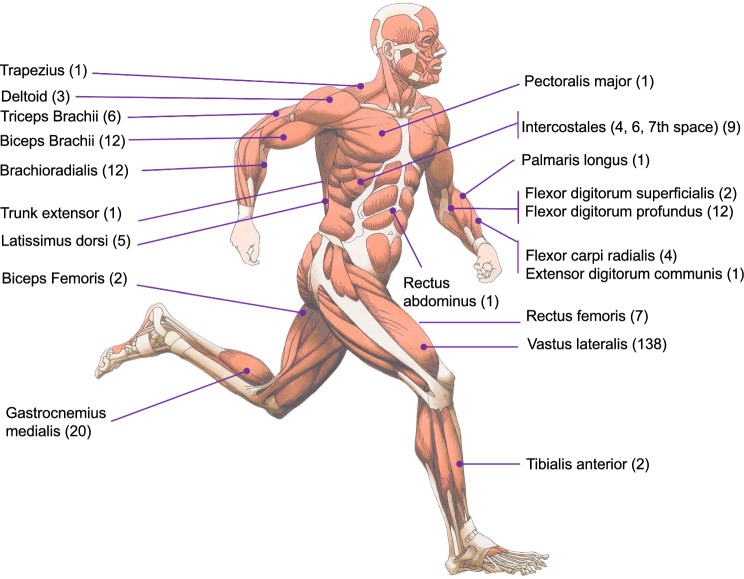
Muscle oxygenation sites measured by muscle oximetry. Number of studies in brackets

A total of 20 muscle sites (Fig. 2), covering both the lower and upper body, were measured by muscle oximetry during exercise. The vastus lateralis muscle (*n* = 138) was used in the majority of the 191 studies for the lower limbs, and the biceps brachii and brachioradialis muscles (*n* = 12 each) were used for the upper limbs. The adipose tissue thickness was not measured in 128 out of 191 studies using skinfold caliper or ultrasound or computed tomography (CT) or magnetic resonance imaging (MRI).

A range of commercially available muscle NIRS oximeters (Table 2) were used to assess muscle oxygenation during exercise in the 191 selected studies, during either local (small muscle mass) or global (whole body mass) exercise with increased load (isometric or dynamic contraction) on the primary active muscles. A total of 130 studies (68%, Table 1) used wearable devices to assess muscle oxygenation during exercise. Among these studies, a total of 40% used the PortaMon device (Artinis Medical System, the Netherlands) and a total of 24% studies used the Moxy device (Fortiori Design LLC, USA). Both are CW-NIRS wearable systems with wireless data transmission. The remaining 12 NIRS devices (Table 2) utilized different techniques, and several are no more commercially available. In most of the studies (*n* = 179) CW-NIRS was employed. The other two NIRS techniques were FD-NIRS [47, 56, 61, 62, 65, 66, 148, 149, 160, 197] or TD-NIRS [40]. These latter technologies could measure the O_2_Hb and HHb in absolute quantitative units in micromoles/litre, as well as the tHb.

**Table 2:** Commercial muscle NIRS oximeters utilized in the sports science articles included in Table S1

Device	Manufacturer	Technique	Light source	No. of wl (SD, mm)	Time- resolution (Hz)	No. of channels	Measurable parameters	Website
Oxymon MkIII	Artinis Medical Systems, Elst, The Netherlands	Multi-distance CW	Laser diodes	2 (50)	250	1	ΔtHb/ΔO_2_Hb/ΔHHb	http://www.artinis.com
PortaLite^a,b,c^	Artinis Medical Systems, Elst, The Netherlands	Multi-distance CW	LEDs	2 (40)	50	1	TSI, ΔtHb/ΔO_2_Hb/ΔHHb	http://www.artinis.com
PortaMon^a,b,d,e^	Artinis Medical Systems, Elst, The Netherlands	Multi-distance CW	LEDs	2 (40)	10	1	TSI, ΔtHb/ΔO_2_HbΔHHb	http://www.artinis.com
Hb11, Hb14^f^	Astem, Kyoto, Japan	Multi-distance CW	LEDs	2 (3)	1	1	StO_2_/ΔtHb/ΔO_2_Hb/ΔHHb	http://www.astem-jp.com
Humon Hex^a,b,g,h^	Dynometric, Inc, Cambridge, USA	Multi-distance CW	LEDs	2 (2.4)	4	1	SmO_2_	http://www.humon.io
Moxy^a,b,i,j,k^	Fortiori Design LLC, Hutchinson, MN, USA	Monte Carlo modeling/ CW	LEDs	4 (25)	2	1	SmO_2_, autHb	http://www.moxymonitor.com
NIRO-200^l^	Hamamatsu Photonics K.K., Hamamatsu City, Japan	Multi-distance CW	Laser diodes	3 (40)	20	2	TOI, ΔtHb ΔO_2_Hb/ΔHHb	http://www.hamamatsu.com
NIRO-200NX^m^	Hamamatsu Photonics K.K., Hamamatsu City, Japan	Multi-distance CW	Laser diodes	3 (40)	20	2	TOI, ΔtHb/ΔO_2_Hb/ΔHHb	http://www.hamamatsu.com
TRS-20^g^	Hamamatsu Photonics K.K., Hamamatsu City, Japan	TRS	Laser diodes	3 (30)	0.5	1	StO_2_, atHb aO_2_Hb, aHHb	http://www.hamamatsu.com
Imagent	ISS Inc, Champaign, IL, USA	FD	Laser diodes	2	50	16–512	ΔtHb, ΔO_2_Hb, ΔHHb	http://www.iss.com
OxiplexTS	ISS Inc, Champaign, IL, USA	Multi-distance FD	Laser diodes	2 (40)	50	2	SO_2_, atHb, aO_2_Hb, aHHb, reduced scattering coefficient	http://www.iss.com
moorVMS-NIRS	Moor Instruments Ltd, Axminster, UK	Multi-distance CW	LEDs	2 (50)	5	2	SO_2,_ auO_2_Hb auHHb	http://www.moor.co.uk
NIMO^j,^^n^	Nirox Srl, Borgosatollo (BS), Italy	Multi-distance CW	Laser diodes	3 (30)	40	2	SmO_2_, atHb, aO_2_Hb, aHHb	http://www.nirox.it
BOM-L1TRW	Omegawave, Inc., Fuchu, Japan	Multi-distance CW	Laser diodes	3	1	1	StO_2_/autHb/auO_2_Hb/auHHb	http://www.omegawave.co.jp

*a* absolute, *au* arbitrary units, *Δ* relative changes*, tHb* total haemoglobin concentration changes, *CW* continuous-wave spectroscopy, *FD* frequency-domain spectroscopy, *HHb* deoxyhaemoglobin concentration changes, *LED* light-emitting diode, *NIRS* near-infrared spectroscopy,* O*_*2*_*Hb* oxyhaemoglobin concentration changes, *SD* longest source–detector distance, *SmO*_*2*_/*SO*_*2*_/*StO*_*2*_/*TOI*/*TSI* muscle oxygen saturation %, *TRS,* time-resolved spectroscopy*, wl* wavelengths

^a^Wearable NIRS system with wireless data transmission

^b^Smartphone-controllable system

^c^System no longer commercially available replaced by PortaLite MKII which samples six channels at 100 Hz

^d^Accelerometer is available on request

^e^System no longer commercially available replaced by; (a) PortaMon MkIII which samples six channels at 100 Hz, and exports six channels at 100 Hz; (b) Train.Red FYER (smartphone controllable wearable waterproof to IPX7 system with wireless data transmission, and accelerometer; https://train.red/) which samples and exports one channel at 10 Hz; (c) Train.Red PLUS (smartphone-controllable wearable waterproof to IPX7 with wireless data transmission, and accelerometer; https://train.red/) which samples six channels at 100 Hz, and exports one channel at 10 Hz [236]

^f^Hb11 system no longer commercially available replaced by Oxy-Pro (Hb141; smartphone-controllable wearable sweat-proof 20 Hz system with wireless data transmission, accelerometer, and fat layer correction)

^g^System no longer commercially available

^h^Waterproof to IPX7

^i^Waterproof to IPX8

^j^Fat-layer correction

^k^Water spectra with fixed water volume fraction included in the calibration model

^l^System no longer commercially available replaced by NIRO-200NX

^m^Not commercially available in European Union

^n^Measured water absorption used to calculate optical path length

### Main Findings

Over the last 5 years, there has been a significant growth in the use of NIRS technique to study muscle oxygenation in conjunction with physical exercise and training as an intervention (Table 1). Regarding the sample size, in a total of 52 studies 20 and more participants were recruited, and in a total of 139 studies less than 20 participants were recruited. Several NIRS use and applications in sport sciences research can be found among the 191 studies reported in detail in Table S1.

#### Variables in Evaluating Muscle Oxygenation

The variables reported in the review for evaluating muscle oxygenation are:

1.NIRS-derived muscle O_2_ saturation (rSO_2_/SmO_2_/StO_2_/SO_2_/TOI/TSI named according to the different instruments);

2.muscle O_2_ consumption (mVO_2_), evaluated by measuring changes in (O_2_Hb + O_2_Mb) during arterial occlusion manoeuvres;

3.blood flow changes evaluated as tHb changes;

4.absolute blood flow evaluated by using venous occlusion manoeuvres;

5.the rate of reoxygenation after submaximal, maximal and brief high-intensity exercise.

Absolute blood flow was measured in a total of 35 studies. Specifically, 6 studies assessed muscle blood flow and mVO_2_ [41, 117, 138, 167, 169, 190], while 8 studies measured muscle blood flow [18, 41, 90, 117, 138, 167, 169, 190], and 25 studies quantified mVO_2_ [26, 39, 41, 53, 54, 58, 82, 98, 102, 116, 117, 121, 126, 132, 134, 138, 167, 169, 175, 184–186, 190, 195, 201]. Finally, the rate of reoxygenation after submaximal, maximal and brief high-intensity exercise are among key indicators for assessing muscle oxidative capability [26, 27, 95, 132, 138, 144, 178, 185, 195, 201].

#### Delineating the Exercise Intensity Domains

Specific physiological breakpoints have been assessed using NIRS technique in many muscle groups [73, 79, 91, 115, 145, 179] during ramp incremental exercise testing to better understand how distinct working muscles are differently affected by exercise intensity or training dose. These data were compared with other well-known physiological markers that delineate exercise intensity zones, such as the measurements of the blood lactate [73, 79] and ventilatory [91, 115, 145, 179] thresholds.

#### Examining the Impact of Ergogenic Aids on Muscle Oxygenation Responses

Over the last 5 years, the NIRS technique has received increasing attention in the world of sports and exercise science as a way to examine the physiological effects that different various ergogenic aids may have on changes in muscle oxygenation (e.g. as a means of enhancing muscle performance or recovery). The following situations were compared with some control scenarios to evaluate the muscle oxygenation responses and the potential benefits of the tested product or intervention: the use of a tracksuit jacket with heating elements [162], nonivamide-nicoboxil cream [180], sports compression garment (*n* = 3 for lower limb: tights, calf sleeve, socks; *n* = 1 for upper limb, forearm) [18, 19, 31, 107], core and skin cooling [136], undergoing thigh cooling by a water-circulating pad [85] and oral supplementation with dietary inorganic nitrate-rich and placebo beetroot juice [32, 33, 40, 43, 63, 75, 76, 131, 146, 158, 183, 190, 201], supplementation with pomegranate extract and co-supplementation with *N*-acetylcysteine [39], protein powder [134], pre-exercise ingestion of a drink with higher dissolved O_2_ [135], anthocyanin-rich New Zealand blackcurrant supplementation [27], 1 h after a single dose of mango leaf extract rich in mangiferin and lecithin, or mango leaf extract rich in mangiferin and quercetin, phospholipids addition [53–55], red spinach extract [121], citrulline drink/citrulline malate [190, 196], l-arginine [141, 143], Montmorency cherry polyphenols [67], eicosapentaenoic acid and docosahexaenoic acid (fish oil) [198], caffeine intake [189] and dark chocolate [81].

The extent of notable changes in the muscle oxygenation levels measured with NIRS indicators in response to various physiological stressors (hypoxia, exercise intensity, exercise profile, training) was investigated in the following cases: repeated sprint training [14, 15, 34, 107, 157, 165, 168, 172, 188, 191, 192], high-intensity interval training [15, 25, 43, 137, 157, 164, 184], voluntary hypoventilation at low lung volume versus normal breathing [17, 34, 88, 89, 151], inspiratory muscle training/pre-activation [45, 97, 100, 112, 113, 140, 152, 181, 187], blood flow restriction [12, 14, 147, 149, 191, 192], intermittent bilateral cuff inflation of lower limbs with three 5/10-min ischemia–reperfusion cycles [53], hypoxia (normo- and hypo-baric) [14, 35, 59, 72, 76, 77, 90, 108, 123, 129, 130, 137, 142, 148–150, 168, 188, 191, 192, 200], ischemic preconditioning [38, 46, 57, 111, 112, 120, 138, 153, 169, 172, 173, 199], water immersions [21, 85, 94, 95], breathing atmospheric air at 1.35 atmospheres [94, 95], muscle heating [46], cold face immersion while breath-holding [93], active passive recovery at different air temperature (20–40 °C) and/or simulated altitudes (400–3800 m) [86, 166], photobiomodulation therapy [105], hyperoxic conditions/preconditioning [11, 56, 95, 165, 194], muscle electrical stimulation [138, 144, 173, 184, 197] and stretching [193].

## Discussion

This updated systematic review of studies on the application of muscle oximetry in sports sciences published over the last 5 years, aimed at synthesizing data on the use of muscle oximetry in evaluating oxidative skeletal muscle performance in 37 major sporting disciplines from included 191 studies.

### Methodological Considerations

Some points need to be made about the NIRS devices and measured NIRS variables used in the studies included in this systematic review. Table 2 presents the 14 models of NIRS devices/instruments, belonging to nine manufacturers, used in the 191 selected studies. Only three CW-NIRS instruments (Humon Hex, Moxy and PortaMon), dedicated to muscle measurements only, are lightweight, compact and wearable and have wireless data transmission. The reliability and validity of these oximeters have been reported [203–205]. A study comparing SmO_2_ measurements from Moxy and PortaMon devices provided physiologically credible SmO_2_ measures at rest and during exercise [206]. However, absolute values obtained during exercise were generally not comparable between devices unless corrected by physiological calibration after arterial occlusion. This indicates that further efforts should be made to standardize all muscle oximeters, for example, by using tissue-simulating phantoms [207] and following the guidelines of ISO 80601-2-85:2021 (Medical electrical equipment—Part 2–85: particular requirements for the basic safety and essential performance of cerebral tissue oximeter equipment). The cumbersome instruments, developed for brain oximetry measurements, can be utilized for muscle studies. The light sources used are laser diodes (in eight devices) or light-emitting diodes (LEDs) (in six devices), with the latter being much less expensive. Only one wireless instrument (PortaMon) includes a six-axis motion sensor, a built-in gyroscope and accelerometer, that can be used to acquire real-time position and orientation movement data and synchronize it with NIRS data. To the best of our knowledge, data from the motion sensor have never been included in a publication. This monitoring of movement combined with machine learning may be useful outside the laboratory for both athletes and coaches in sports applications such as performance enhancement, technical analysis and injury risk mitigation [208]. In addition, movement, or any compression on the area near the NIRS optodes may cause changes in local blood flow that are reflected in the NIRS signals. Therefore, some adaptive filtering with this additional reference signal can be used to control any non-physiological alterations in the NIRS signals that may interfere with proper interpretation.

Most of the instruments (*n* = 13) are capable of measuring SmO_2_ and ~ 85% of the studies reported SmO_2_ measurement or equivalent obtained by spatially resolved spectroscopy method aiming to correct for light scattering. One study utilized a TD-NIRS oximeter, while nine studies utilized an FD-NIRS oximeter. FD-NIRS in skeletal muscle is less sensitive to superficial layer while TD-NIRS can disentangle scattering from absorption information. As a result, both methods improve the accuracy of the muscle haemodynamics assessment. The other CW-NIRS studies, assuming constant path length, potentially lead to errors in estimating of muscle metabolic changes due to incorrect assumptions about tissue scattering. This in turn underestimates actual muscle oxygenation/deoxygenation as compared with measurements obtained by real-time path-length determination by either TD-NIRS or FD-NIRS [209, 210]. Therefore, the implementation of TD- and FD-NIRS approaches on dedicated wearable sensors remains to be achieved. This may be an evolving topic for the next generation of NIRS devices.

It is well known that the relatively high attenuation of the near-infrared light in muscle measurements is due to (i) the two main chromophores (Hb and Mb); (ii) light scattering; and (iii) other molecules (mainly skin melanin, water, lipids of the adipose tissue, intramuscular lipids and cytochrome c oxidase) [2]. Adipose tissue greatly attenuates the signals; correcting for its attenuation has been suggested on the basis of the strength of the relationship between NIRS-derived measurements and the adipose tissue thickness [211, 212]. It remains difficult to discriminate between Hb and Mb spectra as they are very similar in the near-infrared range [4]. Therefore, the contribution of Mb desaturation to the NIRS signal during exercise remains unclear [213]. Subjects with darker skin tones have significantly larger and more concentrated melanosomes, which increase the absorption cross-section of melanin, resulting in enhanced light absorption. Some of the studies included in Table S1 reported the impossibility to perform oximetry measurements on subjects with a dark pigmented skin. More recently, the robustness of two commercial CW-NIRS oximeters (using four or five wavelengths) to variations in skin pigmentation was evaluated using a tissue-simulating phantom; unexpectedly increasing melanin content decreased the O_2_ saturation values displayed by both devices [214]. Differences in melanin content must be taken into account when measuring SmO_2_ values [215]. In addition to the topics included in Table 3 entitled “Pressing issues to improve the quality in muscle oximetry for sports science” of our 2018 review [4], we suggest reporting skin colour using the Fitzpatrick skin type classification scale.

### Meaningful and Promising Applications of Muscle Oxygenation Measures

Monitoring muscle oxygenation during exercise, as assessed by wearable NIRS, is becoming a common physiological marker of internal burden [9]. Muscle O_2_ saturation measured by wearable NIRS was found to have similar reliability to O_2_ uptake and heart rate, across exercise intensities, suggesting that it is suitable for daily use as a non-invasive method of monitoring internal burden alongside other regular systemic physiological variables [216]. Thus, NIRS can become one of the primary monitoring tools in practice, such as heart rate or mechanical power monitoring during endurance exercise. Muscle oxygenation offers a distinct viewpoint on the physiological response of the muscle site being investigated in conjunction with, or in comparison with, other systemic physiological responses observed in different exercise contexts and through different types of training. Currently, wearable NIRS measurements using either PortaMon or Moxy, demonstrated moderate-to-excellent relative reliability scores, and CV as low as 10% for SmO_2_ [217, 218]. Hence, NIRS is a functional tool in which skeletal muscle oxygenation data can be viewed in ‘real time’, complementing external power and heart rate data, allowing coaches to make more informed decisions [4]. Real-time potential can guide athletes’ muscle performance during training and competition, by providing real-time feedback on the metabolic status of the working muscle groups during exercise. This information can be advantageous for handling large datasets by utilizing machine learning models in predicting internal burden [219] and may be suitable for diagnosing muscle fatigue during long-term monitoring. There is probably also a need to establish individual reference values of muscle oxygenation during standardized tests, such as during incremental exercise to exhaustion and an isometric contraction test inducing total arterial occlusion. These two tests performed regularly according to the training programme make it possible to assess the relative evolution of the metabolic state of the trained muscles.

VO_2_max, ventilatory/lactate thresholds and maximum lactate steady state are basic physiological evaluations related to endurance performance. The minimum level of oxygenation and the magnitude of O_2_ extraction in the biceps brachii, latissimus dorsi and vastus lateralis muscles were found to be more predictive of canoe kayak performance than VO_2_max [156] in a short endurance event (200 m). While measuring muscle oxygenation appears to be useful, this finding needs to be replicated and tested in other sports involving different muscle groups. Submaximal to maximal exercise intensities are prescribed to optimize training and improve cardiovascular fitness and endurance using specific intensity zones (moderate, heavy and severe). Typically, this is done by identifying ventilatory or blood lactate thresholds, and critical power/speed can be used. The determination of thresholds or zones of exercise intensity domains using muscle oxygenation variables as an alternative to pulmonary gas exchange or blood lactate methods has been extensively tested in the last 5 years [73, 79, 91, 115, 145, 179]. However, the proximity of the deoxygenation breakpoint to the respiratory compensation point remains controversial [220–222]. Yogev et al. [222] reported that the deoxygenation breakpoint derived from a wearable NIRS sensor over the vastus lateralis did not differ from the respiratory compensation point in a group of trained male and female cyclists with heterogenous fitness. A recent systematic review and meta-analysis on 15 studies indicated that the reliability (based on intraclass correlation coefficient) between the first ventilatory or lactate threshold and the first muscle oxygenation threshold was 0.53 (based on data from 3 studies), while the second threshold was 0.80 [223]. These moderate to good reliability values for the determination of the second ventilatory and lactate thresholds with the NIRS device are likely due to significant variations between the methods of determination, the ability to detect the first threshold and other factors to be investigated (e.g. muscle region, adipose tissue influence). This appears similar to the decades-long debate surrounding the ventilatory profile reported with two inflection points during graded exercise testing [224]. Concerns about using these indices interchangeably are raised by the considerable individual variability. Apart from the measurement of metabolic breakpoints, contextual observations of muscle oxygenation responses and their repeatability may still provide practitioners with pertinent data to comprehend a specific athlete's response to endurance exercise. Muscle oxygenation provides athletes with a targeted measurement of muscle performance during exercise.

An approach to appraise the determinants and limitations of endurance exercise performance is by identifying the work rate that corresponds to the highest steady-state metabolic rate. The critical power/speed model, and the work rate at maximal lactate steady state are widely accepted approaches for this purpose. Recent research [225] has found that, during whole-body exercise, dynamic muscle oxygenation profiles which describe the balance between muscle O_2_ supply and metabolic O_2_ demand are a valuable physiological surrogate for critical metabolic rate. This refers to the highest exercise intensity at which a plateau in the muscle oxygen saturation rate (zero-slope) is reached [224]. Critical oxygenation, as an alternative to critical power/speed, may provide insight into the causes of muscle performance and fatigue in various sporting activities, through its determination at different workloads and durations, based on a physiological framework [226]. It is worth noting that critical metabolic rate, determined by the balance between muscle O_2_ supply and metabolic demand in quadriceps and forearm muscle sites, predicts the time of exhaustion during continuous and intermittent exercise [227].

Deoxygenation breakpoint measurements [222, 228] may allow better categorization of the training stimulus into zones of exercise intensity (e.g. percentage of one-repetition maximum for the load prescription or percentage of maximum voluntary contraction) for specific muscle groups in resistance/strength training, thereby favouring the desired local muscle adaptations. Strength training research is frequently interested in how and when muscle is activated/recruited during movement, when muscle fatigue occurs and how different neuromuscular mechanisms contribute to force production. Surface electromyography (sEMG) is the most commonly used measurement for these purposes. However, evaluating the effects of resistance training on skeletal muscles might be characterized by a lower muscle oxygenation response due to a restriction in blood supply to the primary muscle in relation to the number of repetitions and the load, which induces increased intramuscular mechanical pressure. A recent systematic review [229] examined baseline and end-points values acquired by NIRS during resistance exercise in healthy persons. SmO_2_, the most studied variable with NIRS devices (Moxy and Portamon), decreases as an acute response to muscular strength exercise, according to the four included lower limb studies using squat-like exercise modalities.

Another relevant variable to discuss is that in some selected articles we have observed that data reported by NIRS during exercise or training interventions have been introduced by adding manipulations in the form of venous and arterial occlusion to assess both muscle blood flow and mVO_2_ [41, 117, 138, 167, 169, 190]. Muscle oxidative capacity is the maximum rate at which the muscle can utilize O_2_ to meet the energy demand of exercise [230]. With NIRS, the initial rate of muscle deoxygenation during transient arterial occlusion is a direct measure of mVO_2_, a reliable indicator of muscle oxidative capacity [2]. In the present review, 25 studies quantified mVO_2_ (see Table S1 and Results). Venous occlusion has also been used with NIRS to provide measurements of muscle blood flow during or after repeated high-intensity exercises [231]. Incorporating this simple test both before and after intervention [232] provides additional insight into changes in muscle haemodynamics and metabolic activity as a result of training. During and after exercise, repeated transient arterial occlusions can yield sequential mVO_2_ measurements [82, 232]. Therefore, for the purpose of determining changes in mitochondrial capacity, the recovery of mVO_2_ values after exercise provides information that is essentially the same as that obtained from the kinetics of phosphocreatine levels after exercise [233, 234]. These possibilities offered by NIRS may be useful for practitioners.

The NIRS tests, evaluating potential physiological responses that may contribute in some way to the ergogenic effects, are the final aspect of the data collected in the present review. Coaches and athletes are looking for effective strategies to enhance performance and speed up recovery. The NIRS device was employed to find out whether the ergogenic aid would affect the balance between local O_2_ delivery and muscle O_2_ utilization in skeletal muscle and thus improve performance. Ergogenic aids are any of the methods, including dietary, pharmacological and physiological ones, that can improve performance. Cherry polyphenols [67] and mango leaf extract rich in mangiferin [53–55] may be useful nutritional aids for improving muscular endurance performance. Contrarily, this does not appear to be the case for acute dietary nitrate supplementation [33, 63, 75, 76, 158, 183, 190], citrulline [190, 196], l-arginine, red spinach extract [121], dark chocolate [181] and caffeine [189]. Additionally, mechanical ergogenic aids like compression clothing [18, 19, 31, 107] tend to increase blood flow and muscle oxygenation, especially at rest. With regard to physiological ergogenic aids, techniques like blood flow restriction [12, 14, 147–149, 191, 192], voluntary hypoventilation at low lung volume [17, 34, 88, 89, 151], electrical muscle stimulation [138, 144, 173, 184, 197] and dynamic stretching [193] typically alter the muscle oxygenation response; however, the effects of other interventions like photobiomodulation therapy [105] or immersion in water [21, 85, 94, 95] are less certain. All these data, obtained using different protocols, show that the sensitivity of the NIRS technology is being study here to assess the true value of a particular ergogenic aid. It is interesting to note that they show how some ergogenic aids can modulate blood volume (strictly related to tHb) variations and hence the balance between O_2_ delivery and utilization (and hence O_2_ extraction) within the interrogated region.

### Unanswered Questions and Future Research

Because the current NIRS technique only allows for the analysis of a small volume of muscle (superficially, with the average depth reaching only half the distance between the light source and the detector, e.g. 1.5–3 cm approximately), it is currently not indicative of what is happening in the other working muscles. We may develop NIRS equipment that can be integrated into sports clothing and can be utilized outside of the laboratory using energy harvesting technologies (solar batteries, sunlight as a light source, and bandpass filters). This equipment would be used to map whole-body activity. The surface electromyogram sensors should be utilized in conjunction with the NIRS equipment to provide an overall picture of changes in muscle function during exercise [235]. In addition to the haemodynamics and metabolic data provided by NIRS, we also gain knowledge about muscle-level activation using surface electromyography, and the effect of motion from accelerometers on these two signals.

### Limitations and Strengths

Although this review was conducted according to the PRISMA guidelines updated in 2020 [10] and with standardized critical appraisal, several factors limit our ability to draw strong conclusions.

A common limitation of all systematic reviews is that some articles may be overlooked. To overcome this problem, we conducted an extensive search using sensitive search criteria and synonyms. Another limitation of this systematic review is that the quality of the study varied widely. For instance, details of participant selection were unclear in most articles. There was a large heterogeneity in the study populations examined. The number of participants was generally small (*n* < 20 in 72% of the studies), which limits the generalizability of the obtained results. Several studies did not follow a standardized research protocol. There was also considerable heterogeneity in study design and outcomes.

While the included studies mainly focused on athletes at the national/international level, differences between the included sports in terms of training, remuneration and other relevant parameters need to be taken into account. Although systematic reviews are generally considered to be of as “a high quality of evidence”, we believe that the reported findings are of moderate quality, taking into account the limitations mentioned above. A strength of the present review is that it provides an update of our previous systematic review of the literature on muscle oximetry in sports science [4]. Given the heterogeneous nature of the reported studies and the wide variation in the methods used, it is not possible to draw general conclusions about the role of muscle oximetry in sports science.

## Conclusions and Prospects

There is no doubt that NIRS is a useful method for evaluating muscle adaptation effects in studies involving intermittent or continuous aerobic/anaerobic exercise and strength training, and it can therefore be utilized by physical trainers to guide training or recovery processes, and to test many potential interventions (the so-called ergogenic aids) favouring changes in the balance of O_2_ delivery and O_2_ utilization as a key factor in muscle performance.

The future of muscle oximetry in sports science is closely related to the instrumental development. In terms of miniaturization, CW-NIRS technology is the most convenient method. Different lightweight (about 20 g), compact, smartphone-controllable and wearable multi-distance CW-NIRS-based oximeters with Bluetooth connection up to 150 m and on-board data collection (up to 50 h) are commercially available [236]. The novel CW-NIRS oximeter (Train.Red Plus, Artinis Medical Systems) also includes very useful haptic feedback, so that the subject can feel a buzzer on the skin during the exercise [237]. The CW-NIRS technology has been incorporated into OctaMon M (Artinis Medical Systems), the only commercial imager dedicated to muscle studies (50 Hz sampling time; four source–detector distances in the range 25–40 mm) using eight measurement points [238].

Multi-modal techniques based on two types of sensors, sEMG and CW-NIRS, have been developed to provide a more detailed evaluation of muscle activity, as the information obtained by each sensor is based on different phenomena induced by muscle activity. The fusion of these wearable technologies in sporting garments can provide an objective assessment of the quality and the quantity of the muscle activity, as well as the continuous monitoring of exercise programs. A new wearable integrated quadriceps muscle oximetry/sEMG system adopting smart textiles for sEMG has been recently tested under resting and dynamic conditions (treadmill running and resistance exercise) [239]. More recently, the sEMG and CW-NIRS signals have been measured during isometric ramp contraction of the forearm and cycling exercise of the vastus lateralis muscle with stepped increments of the load using a wireless multi-layered sensor [240]. More complex devices using FD-NIRS and TD-NIRS allow the monitoring of both absorption and scattering and can provide more accurate signals under a wider range of conditions [2, 235]. The first commercial FD-NIRS and TD-NIRS systems were introduced in 1998 and 2003, respectively, but their high price limited the application. Recently, a commercial two-wavelength battery-operated wireless wearable TD-NIRS system, which fits into a backpack (3 kg) and performs measurements on the brain and muscle tissue of freely moving subjects using a 10-g optode, has been introduced [241]. This system provides SmO_2_ as well as absolute concentrations of O_2_Hb and HHb at 20 Hz.

Over the last 20 years, diffuse correlation spectroscopy (DCS) has emerged as a versatile, non-invasive method for the continuous measurement of microvascular blood flow as a tissue blood index [242]. DCS uses the temporal fluctuations of diffusely reflected light to quantify the motion of tissue scatterers (primarily the velocity of red blood cells). Application to ramp-incremental cycling exercise has been successfully demonstrated utilizing a complex and expensive commercial hybrid system equipped to employ FD-NIRS and DCS [243]. The technical limitations of this hybrid technology remain an important barrier to wider adoption.

NIRS technology continues to evolve, and the nature of this approach provides distinct advantages when studying human muscle during exercise. Despite current limitations, which are largely confined to limited penetration depth, low spatial resolution and interference from adipose tissue thickness [1], we believe that the feasibility and success of applying muscle oximetry in sports science have been well documented and encourage its routine use in sports science and medicine [4, 6, 7, 9].

### Supplementary Information

Below is the link to the electronic supplementary material.

Supplementary file1 (PDF 591 KB)

## Declarations

### Data availability

All data and material reported in this systematic review are only from peer-reviewed publications. All the extracted data were included in Table S1 of the ESM, Tables 1 and 2, and in Fig. 2.
