# Supplementary material for: Muscle Oximetry in Sports Science: An Updated Systematic Review
Source: Sports Med. 2024 Feb 12;54(4):975–96. doi: 10.1007/s40279-023-01987-x (PMC11052892; doi:10.1007/s40279-023-01987-x)
Supplement: Supplementary file 1 — Supplementary file1 (PDF 591 KB) [file 40279_2023_1987_MOESM1_ESM.pdf]

Electronic Supplementary Material Table S1

Perrey S, Quaresima V, Ferrari M. Muscle oximetry in sports science: An updated systematic review. Sports Medicine (2024) Volume 54

DOI: 10.1007/s40279-023-01987-x

Table S1 Summary of findings for studies investigating muscle tissue oxygenation in moderately trained to elite athletes during and after various sports carried out on the field or simulated in laboratory settings[a]

Sport	Study (year)	Sample	Participants[b]	Exercise, protocol	Instrumentation and measured NIRS variables[c]	Muscle(s)	Results
**Alpine skiing**	Morawetz et al. (2020) [11]	17	Highly skilled skiers (6 F, 27.2 ± 1.3 years, 63.2 ± 6.5 kg; 11 M, 29.0 ± 5.7 years, 69.1 ± 7.1 kg)	60 s constant-load, all-out cycling test in a normobaric hypoxic chamber with hyperoxic preconditioning vs. non hyperoxic preconditioning	Moxy[d] (SmO_2, tHb)	Right vastus lateralis (Sf: NA)	No effect of hyperoxic preconditioning on muscle de- and re-oxygenation during and after the all-out test. No tHb change during the all-out test
**American football**	Fortin and Billaut (2019) [12]	16	Laval University Canadian football team (M: 21.0 ± 1.8 years, 82.1 ± 2.8 kg).	Repeated-sprint ability test (12 × 20 m, 20 s rest), preceded by blood-flow restriction or a regular warm-up	Moxy[d] (SmO_2, tHb)	Bilateral gastrocnemius (Sf: 6.9 ± 1.7 mm)	Improved muscle hemodynamics during a repeated-sprint ability test with blood-flow restriction
**Badminton**	Girard et al. (2020) [13]	8	National team badminton players (4 F/4 M: 21.3 ± 2.7 years, 70 ± 10 kg)	Badminton-specific repeated-agility test	PortaMon[d] (TSI)	Vastus lateralis (Sf: NA)	No effect of ischemic pre-conditioning during recovery period between successive doubles badminton on TSI trends
	Valenzuela et al. (2019) [14]	8	Elite badminton players (M: 20 ± 2 years, 71 ± 4 kg)	3 on-court repeated-sprint training sessions (3 sets of 10 repetitions of 10-s badminton-specific movements in normoxia), systemic normobaric hypoxia (FiO_2 14%) or with mBF restriction on the thighs (40% arterial occlusion pressure)	Moxy[d] (SmO_2, tHb)	Vastus lateralis (Sf: NA)	Lower muscle oxygenation with repeated-sprint training, but no differences in muscle oxygenation between conditions
**Basketball**	Delextrat et al. (2018) [15]	20	Elite junior basketball players (M: 14.3 ± 0.5 years, 74.5 ± 9.8 kg)	30-15 intermittent fitness test and repeated sprint sequence (2 bouts of 15 s all-out sprint) pre- and post- training interventions (high-intensity interval training and small-sided games)	PortaMon[d] (TSI, ΔO_2Hb, ΔHHb, ΔtHb)	Vastus lateralis (Sf: NA)	Greater TSI changes and improvements in reoxygenation after both training interventions

	Guan et al. (2021) [16]	10	University basketball players (M: 20 ± 1 years, 87.6 ± 7.6 kg)	4 groups of exercise, consisting of 3 sets of 10 loaded counter-movement jumps with a 30% 1 repetition maximum load (1-, 2-, 3-, or 5-min recovery times)	PortaMon[d] (TSI, ΔO_2Hb, ΔHHb, ΔtHb)	Left vastus lateralis (Sf: NA)	No difference between sets and groups for TSI baseline values, the lowest values after jumps for each set, the decline in O_2Hb and TSI, and re-oxygenation during recovery periods. TSI always recovered to baseline in 1 min
	Lapointe et al. (2020) [17]	17	National level basketball players (5 F/12 M: 22.3 ± 1.2 years, 88.6 ± 16.9 kg)	Before and after training, repeated-sprint ability test (12 × 30-m, 25-s rest) and a 30-15 intermittent fitness test. Training: over 4 weeks 8 sessions of straight-line running repeated-sprint and repeated-sprint with changes of direction, performed either with normal breathing (*n* = 9) or voluntary hypoventilation at low lung volume (*n* = 8)	PortaMon[d] (ΔO_2Hb, ΔHHb, ΔtHb)	Gastrocnemius lateralis (Sf: NA)	Voluntary hypoventilation lowered maximal ΔHHb induced by sprints and enhanced reoxygenation during recovery periods
	O'Riordan et al. (2023) [18]	22	Elite basketball players (M: 17.2 ± 0.9 years, 90.0 ± 9.9 kg)	Resting lower-limb mBF in 4 conditions: no compression, compression tights, compression shorts, compression socks	OxyMon MkIII (TSI, ΔO_2Hb, ΔHHb, mBF)	Gastrocnemius medialis, vastus lateralis (Sf: NA)	Higher gastrocnemius medialis mBF for compression tights compared to control, compression shorts, and compression socks, with compression socks higher compared to compression shorts. Higher vastus lateralis mBF for compression tights compared to control and compression socks, with compression shorts also higher compared to control and compression socks.
	Paulauskas et al. (2020) [19]	12	Highly trained basketball players (M: 21 ± 1.9 years, 86.2 ± 5.8 kg)	10 x 30 m sprints interspersed with 30 s of passive recovery; 20 x 15 m shuttle sprints interspersed with 15 s of passive recovery. Both exercises repeated 3 times	Moxy[d] (SmO_2, tHb)	Dominant leg vastus lateralis (Sf: NA)	SmO_2 decreased and recovered during passive rest intervals without showing differences in both repeated sprint exercise modalities
**Climbing**	Baláš et al. (2018) [20]	32	Sport climbers (17 F: 26.3 ± 4.6 years, 57.7 ± 5.6 kg; 15 M: 27.7 ± 10.2 years, 71.0 ± 9.3 kg); intermediate to advanced ability level	Exhaustive intermittent handgrip exercise (8 s contraction, 2 s relief) at 60 % MVC on 3 separate days	OxyMon MkIII (TSI, ΔtHb)	Dominant hand flexor digitorum profundus (Sf: NA)	Reliable measures of deoxy- and reoxygenation during intermittent handgrip contractions in rock climbers
	Baláš et al. (2020) [21]	29	Intermediate to advanced ability level rock-climbers	3 repeated intermittent 60% MVC handgrip contractions	OxyMon MkIII (TSI, ΔO_2Hb, ΔHHb, ΔtHb)	Finger flexor digitorum (Sf: F 2.0 ±	During exercise, tHb changes were similar for all 3 recovery strategies in

		(17 F, 26.3 ± 4.6 years, 17 M, 27.7±10.2 years)	(work:relief ratio of 8:2 s) to failure with 20-min recovery strategies (8 or 15 °C cold water immersion or passive recovery)		0.7, M 1.7 ± 0.5 mm)	M and F. Similar deoxy - and reoxygenation after all recovery strategies. Lower TSI during contractions with recovery at 8°C. Drop of TSI to a greater degree with temperature strategies than after passive recovery strategy
Baláš et al. (2021) [22]	22	11 advanced (35.5 ± 7.6 years, 70.9 ± 5.3 kg) and 11 intermediate (27.4 ± 8.2 years, 69.5 ± 6.6 kg) sport climbers. All M	Isometric sustained and intermittent forearm flexor contractions, and an exhaustive climbing test on motorized climbing ergometer	PortaMon[d] (TSI)	Dominant hand flexor digitorum profundus (Sf: NA)	Climbing-specific endurance determined with muscle oxygenation kinetics
Dindorf et al. (2023) [23]	42	Trained climbers or bouldering athletes (22 F/20 M: 22.4 ± 3.1 years, 70.5 ± 12.3 kg)	3 intermittent measurements of climbing-specific holding time by performing dead hangs, and of handgrip strength	Moxy[d] (SmO_2)	Flexor carpi radialis, palmaris longus (Sf: NA)	Acute fatigue objectification using athletes' termination SmO_2. SmO_2 inappropriate for monitoring cumulative fatigue during intermittent isometric climbing-specific muscle contractions
Feldman et al. (2020) [24]	11	Elite climbers (M: 24 ± 4 years, 65.7 ± 2.5 kg)	Finger-hang test under 4 weighted conditions	Moxy[d] (SmO_2)	Flexor digitorum profundus (Sf: NA)	Constant SmO_2 at high intensity conditions and related to time to task failure
Feldmann et al. (2021) [25]	21	Expert climbers (F: 9, 21.8 ± 5.6 years, 53.5 ± 6.7 kg; M: 12, 30.3 ± 7.6 years, 66.8 ± 5.6 kg)	High-intensity training task (four 5-min exhaustive climbing tasks)	Moxy[d] (SmO_2)	Flexor digitorum profundus (Sf: NA)	Potential state of athlete readiness with the change of SmO_2 metrics from training
Fryer et al (2017) [26]	33	Boulderers vs. lead sport climbers (M: 13 advanced boulderers, 27.5 ± 5.7 years, 72.7 ± 6.2 kg; 10 advanced lead climbers, 26.1 ± 5.3 years, 71.1 ± 8.2 kg; 10 controls, 26.7 ± 4.2 years, 74.9 ± 13.5 kg)	Measurement of oxidative capacity index, mVO_2	PortaLite[d] (TSI, ΔO_2Hb, ΔHHb, ΔtHb)	Flexor digitorum profundus, extensor digitorum communis (Sf: boulderers, 3.0 ± 0.6/3.0 ± 0.6; climbers, 4.0 ± 1.0/3.9 ± 0.8; controls, 4.0 ± 1.0/3.9 ± 0.8 mm)	Time to half recovery after handgrip dynamometry exercise greater in both climbing groups. No mVO_2 difference
Fryer et al. (2021) [27]	12	High-level rock climbers (6 boulderers, 6 sport climbers) (M: 25 ± 4 years, 67.9 ± 5.7 kg)	Oxidative capacity assessment, 2 successive exhaustive exercise trials (submaximal forearm muscle contractions at 60% MVC) following 7-days anthocyanin-rich New Zealand	PortaLite[d] (TSI, ΔO_2Hb, ΔHHb, ΔtHb)	Flexor digitorum profundus (Sf: 1.5 ± 0.3 mm)	Greater muscle oxidative capacity with no effect on isolated muscle performance following 7-days intake

				blackcurrant supplementation or placebo			
	Gajdošík et al. (2022) [28]	32	Intermediate and advanced sport climbers (10 F intermediate: 28.5 ± 7.7 years, 61.7 ± 7.4 kg; 11 M intermediate: 27.4 ± 7.8 years, 69.5 ± 6.3 kg; 11 M advanced: 35 ± 7.2 years, 70.9 ± 5.1 kg)	Three 4-min-long ascents of same route at 4, 6, and 9 m/min on motorized climbing ergometer (Treadwall)	PortaMon[d] (TSI, ΔO_2Hb, ΔHHb, ΔtHb)	Flexor digitorum profundus (Sf: NA)	TSI decrease present for intermediate climbers. Distinction of groups during steady state climbing task with TSI rather than systemic O_2 responses. Larger between-group TSI differences with increased climbing speed
	Limmer et al. (2022) [29]	24	Sports climbers (12 F: 28.2 ± 6.2 years. 59.4 ± 5.6 kg; 12 M: 30.0 ± 7.2 years, 74.8 ± 10.2 kg)	Intermittent hand grip strength and endurance measurements, finger hang, and lap climbing while wearing compression forearm sleeves, non-compressive forearm sleeves or no forearm sleeves	OxyMon MkIII (TSI, ΔO_2Hb, ΔHHb, ΔtHb)	Flexor digitorum profundus (Sf: NA)	Slightly more pronounced changes of O_2Hb and TSI in the deoxy- and reoxygenation phases with compression sleeves during the hand grip strength and endurance measurements; tHb not affected
	Nolan et al. (2020) [30]	21	Aerobically trained athletes (M: 6 aerobically trained (23 ± 1 years, 77 ± 1 kg; 7 power lifters (24 ± 1 years, 80 ± 3 kg), 8 rock climbers (25 ± 2 years, 74 ± 2 kg)	Three fatigue protocols: sustained MVC until exhaustion (1); sustained 3-min 40% MVC (2); 3-min intermittent 40% MVC (5-s contraction, 5-s recovery) (3)	Moxy[d] (SmO_2)	Forearm flexor (Sf: NA)	O_2 desaturation half-time response longer in rock climbers versus aerobically trained participants, and associated to a better isometric fatigue resistance
**Combat sport**	Antunes et al. (2022) [31]	15	8 judokas, 7 heterogenous fitness participants (M: judokas, 21.1 ± 3.0 years, 71.5 ± 7.1 kg; controls, 22.6 ± 1.0 years, 64.3 ± 5.8 kg)	Incremental step test: two heavy intensity square-wave transitions and upper body repeated-sprint ability test (four 15 s sprints/45 s rest)	NIMO (aHHb)	Right triceps brachii (Sf: 4.5 ± 0.7 mm, 6.6 ± 1.6 mm)	Incremental test HHb kinetics not associated with increased upper body repeated-sprint ability
	de Oliveira et al. (2018) [32]	12	Jiu-jitsu athletes (M: 29 ± 7 years, 81.3 ± 10.1 kg)	3 sets of handgrip isotonic exercise at 40% MVC until fatigue after 8 days of beetroot-based gel supplementation or placebo	PortaMon[d] (TSI, ΔtHb)	Dominant forearm flexor digitorum profundus (Sf: NA)	Greater TSI during exercise recovery only after beetroot supplementation. No tHb difference
	de Oliveira et al. (2020) [33]	14	Recreational jiu-jitsu and judo athletes (M: 29.9 ± 8.5 years, 79.7 ± 8.5 kg)	3 sets of handgrip isotonic exercise at 40% MVC until fatigue	PortaMon[d] (TSI, ΔtHb)	Dominant forearm flexor digitorum profundus (Sf: NA)	No TSI changes after single oral dose of beetroot-based gel and after placebo

	Woorons et al. (2019) [34]	10	Highly trained Jiu-Jitsu fighters (3 F/ 7 M: 19.2 ± 2.3 years: 68.2 ± 10.6 kg)	2 repeated-sprint sessions, each including 2 sets of 8 x ~6 s back-and-forth running sprints on a tatami: one with normal breathing vs. one with voluntary hypoventilation at low lung volume	PortaMon[d] (TSI, ΔO_2Hb, ΔHHb, ΔtHb)	Vastus lateralis (Sf: NA)	No muscle oxygenation difference between conditions during entire exercise
**Cross-country skiing**	Nyback et al. (2017) [35]	8	Competitive cross-country skiers (5 M: 21.8 ± 2.8 years, 73.5 ± 8.3 kg; 3 F: 20.7 ± 1.2 years, 63.4 ± 5.7 kg)	2 x 6 min submaximal exercise bouts and 1000 m time trial on a treadmill using roller-skis in normoxia or normobaric hypoxia (FiO_2 16.8%)	Moxy[d] (SmO_2)	Left vastus lateralis, triceps brachii (Sf: NA)	No effects of acute dose of dietary nitrate on SmO_2 changes in normoxia and hypoxia
	Øfsteng et al. (2018) [36]	29	Well-trained cross-country skiers (M: 21 with 8 weeks upper-body strength training 3 times/week, 38.2 ± 7.7 years, 78.2 ± 7.6 kg; 8 controls, 27 ± 7 years, 77.0 ± 7.8 kg)	Incremental time to exhaustion test using double poling technique while treadmill roller skiing	PortaMon[d] (TSI)	Latissimus dorsi, triceps brachii, vastus lateralis (Sf < 5.1 mm)	Largest desaturation during the test in latissimus dorsi and triceps brachii whatever the group
	Stöggl et al. (2021) [37]	1	One elite cross-country skier (M: 39 years, 80.4 kg)	Muscle oxygenation monitoring during 90 km Vasaloppet cross-country skiing race	Moxy[d] (SmO_2)	Triceps brachii, rectus abdominus, latissimus dorsi, vastus lateralis (Sf: 5.6, 8.2, 8.6, 3.4 mm, respectively)	Interchangeable pattern of triceps brachii SmO_2 with heart rate. Specific loading and unloading pattern of cross-country skiing in uphill and downhill sections with triceps brachii and vastus lateralis SmO_2. Fatigue patterns reflected in rectus abdominus SmO_2
**Cycling**	Aebi et al. (2019) [38]	17	8 trained cyclists vs. 9 recreationally trained cyclists (17 M: 25 ± 2 years, 71.3 ± 7.7 kg)	4 cycles of 5 min stages of cycling at 1.5 W/kg in four conditions (control, ischemic preconditioning, hypoxic preconditioning, preconditioning combined)	PortaMon[d] (TSI, ΔO_2Hb, ΔHHb, ΔtHb)	Vastus lateralis (Sf: NA)	Amplified oxygenation responses hemodynamic responses (e.g., greater O_2 extraction and changes in blood volume) in trained cyclists
	Batterson et al. (2020) [39]	24	Recreational-to-highly-trained participants (M: 36.8 ± 11.6 years, 76.6 ± 9.8 kg)	25-km time trial time-to-completion	OxyMon MkIII (ΔO_2Hb, ΔHHb, ΔtHb, mVO_2)	Vastus lateralis (Sf: NA)	Time constant for mVO_2 recovery explained 92.7% of trial time-to-completion variance
	Breese et al. (2017) [40]	8	Physically active subjects (M: 24 ± 6 years, 66 ± 5 kg)	Two step cycle tests at a work rate equivalent to 50% of difference between GET and peak	TRS-20 (O_2Hb, HHb, tHb)	Rectus femoris, vastus lateralis,	No differences in HHb kinetics (primary mean response time and amplitude)

			VO_2 over separate 4-day supplementation periods with dietary inorganic nitrate rich and placebo beetroot juice		vastus medialis (Sf: NA)	between the 2 trials at each muscle site. Inverse correlation of the VO_2 slow component reduction after supplementation with the mean increases in HHb and tHb across all muscles
Broatch et al. (2018) [41]	20	Recreationally active participants (11 F: 25 ± 2 years, 62.6 ± 9.5 kg; 9 M: 28 ± 6 years, 83.8 ± 9.3 kg)	4 sets of 10 × 6-s maximal sprints on a cycle ergometer, with and without lower-limb compression tights (24-s recovery between bouts and 2-min recovery between sets)	OxyMon MkIII (ΔO_2Hb, ΔHHb, mBF, mVO_2)	Right vastus lateralis (Sf < 35 mm)	Increased quadriceps blood flow during repeated-sprint exercise with lower-limb compression tights
Brock et al. (2018) [42]	9	Competitive athletes (M: 21 ± 3 years, 77 ± 8 kg)	4 target work trials to assess O_2 kinetics and cycling performance. Trials initiated with either a 12-s all-out start or a self-selected start and preceded by prior severe-intensity (70%Δ) priming exercise or no priming exercise	NIRO-200 (ΔO_2Hb, ΔHHb, ΔtHb)	Vastus lateralis (Sf: NA)	Higher HHb and tHb at baseline and throughout exercise in primed trials. Shorter HHb time constant + time delay in all-out primed trial compared to all other experimental conditions, and in all-out unprimed compared to self-paced unprimed control trial
Broeder et al. (2021) [43]	10	Cyclists and triathlon race athletes (M: 41.4 ± 9.1 years, 83.3 ± 9.6 kg)	High-intensity interval protocol at 50% above functional threshold power with or without oral beet nitrate supplementation	Moxy[d] (SmO_2, O_2Hb, HHb)	Right vastus intermedius (Sf: NA)	During oral beet nitrate supplementation, SmO_2 better maintained throughout entire high-intensity interval protocol
Cayot et al. (2021) [44]	14	Recreationally active cyclists (5 F/9 M: 27 ± 6 years, 77.5 ± 12.8 kg)	Maximal incremental step cycling tests (25 W/3 min) to volitional fatigue on two separate occasions	Moxy[d] (SmO_2, tHb)	Vastus lateralis (Sf: 13 ± 4 mm)	Moderate to good reliability of SmO_2 at moderate (50% peak work rate) and peak (100% work rate) cycling intensities
Cheng et al. (2020) [45]	9	Division I collegiate basket players (M: 21 ± 1 years, 83 ± 11 kg)	Repeated sprint (6 x 30 s interval exercise)	PortaMon[d] (TSI, ΔO_2Hb, ΔHHb, ΔtHb)	Right vastus lateralis (Sf: NA)	ΔtHb lower at rest intervals in inspiratory resistive loading than in control condition; reduced O_2 delivery to active muscles
Cocking et al. (2021) [46]	11	Trained cyclists (M: 33.7 ± 4.8 years, 81.1 ± 10 kg)	All-out repeated-sprint task (10 × 6-s sprints with 24-s recovery) preceded by ischemic preconditioning or sham, combined with passive muscle heating or thermoneutral insulation	PortaMon[d] (TSI, ΔO_2Hb, ΔHHb, ΔtHb)	Right vastus lateralis (Sf: NA)	No muscle oxygenation changes between conditions
Colosio et al. (2021) [47]	8	Trained cyclists (M: 25 ± 2 years, 74 ± 10 kg)	3-, 6- and 9-min constant work rate cycle ergometer exercise in	OxiplexTS (aHHb)	Left vastus lateralis (Sf: NA)	In the 3 exercises, there was a domain-dependent dynamic over time for VO_2 and HHb (taken as an index of

			moderate, heavy, and severe intensity domains, respectively			metabolic instability). About 52 % of VO_2 slow component dynamics explained by HHb
Combes et al. (2018) [48]	12	Trained subjects (M: 24 ± 5 years, 76 ± 10 kg)	Two exercises at 70% peak work rate: one 30-min bout, 30 1-min bouts/1-min recovery	PortaMon[d] (ΔO_2Hb, ΔHHb, ΔtHb)	Left vastus lateralis (Sf: NA)	Despite similar energy expenditure and intensity, HHb fluctuations were higher in intermittent than continuous exercise modalities
Crum et al. (2018) [49]	8	Trained cyclists (6 F/6 M: 37 ± 11 years, 73.0 ± 12 kg)	Submaximal cycling and a 5-min time trial	Moxy[d] (SmO_2, tHb)	Vastus lateralis of each leg (Sf: NA)	No main effect of treatment (pomegranate extract supplementation and co-supplementation with N-acetylcysteine) on SmO_2 and tHb after four 8-day period
Espinosa-Ramírez et al. (2021) [50]	25	Physically active participants (12 F: 21 ± 1 years, 55 ± 3 kg; 13 M: 22 ± 1 years, 68 ± 6 kg)	Ramp incremental test on cycle ergometer (20 W/2 min)	Moxy[d] (SmO_2)	Intercostals and vastus lateralis (Sf: NA)	Greater deoxygenation of respiratory muscles in women, greater deoxygenation of vastus lateralis in men
Fennel et al. (2021) [51]	14	Well-trained high-performance cyclists (14 M: 33 ± 13 years, 70.6 ± 8.1 kg)	6 x 4 min or 3 x 8 min high intensity interval training with 3 recovery intensity prescriptions	PortaMon[d] (TSI, ΔO_2Hb, ΔHHb, ΔtHb)	Vastus lateralis (Sf: 9.5 ± 2.7 mm)	Greater TSI change during passive recovery intervals compared to active recovery at 80% and 110% of lactate threshold
Formenti et al. (2019) [52]	9	Trained cyclists (3 F/6 M: 21-55 years)	Athletes exercised at a power output equal to GET, pedaling at cadences of 40, 50, 60, 70, 80 and 90 rpm, each for 4 min	PortaMon[d] (TSI, ΔO_2Hb, ΔHHb, ΔtHb)	Right vastus lateralis (Sf: NA)	Decreases in TSI from rest to exercise; greater TSI decrease pedaling at 90 rpm compared to pedaling at 40 and 50 rpm
Gelabert-Rebato et al. (2018) [53]	31	Physically active subjects (14 F, 27.0 ± 2.2 years, 56.5 ± 5.4 kg; 17 M, 22.7 ± 2.1 years, 71.2 ± 5.2 kg)	48 h after treatment with placebo, or mangiferin with luteolin, or mangiferin with quercitin and tiger nut extract, two 30-s Wingate tests and a 60-s all-out sprint followed by 20-s tight arterial occlusion. After circulation re-opening, 15-s sprint followed by 10 s recovery; then, another 15-s final sprint	NIRO-200NX (TOI, mVO_2)	Vastus lateralis (Sf: NA)	Better muscular extraction of O_2 in the sprints performed after ischemia/reperfusion when the subjects had taken the combined mango leaf/quercetin/ tiger nut extract
Gelabert-Rebato et al. (2019) [54]	40	Physically active subjects (20 F, 23.5 ± 2.9 years, 59.5 ± 8.0 kg; 20 M, 23.1 ± 2.2 years, 73.4 ± 9.1 kg)	One hour after single dose of mango leaf extract rich in mangiferin and lecithin, or mango leaf extract rich in mangiferin and quercetin, or placebo, three	NIRO-200NX (TOI, mVO_2)	Right vastus lateralis (Sf: NA)	Treatments improved O_2 extraction only in M during the 1st three sprints and in the sprint after 60 s ischemia, as well as muscular performance. No advantage from phospholipids addition

			30-s Wingate tests interspaced by 4 min and final 15-s sprint after ischemia			
Gelabert-Rebato et al (2019) [55]	12	Physically active subjects (M: 21.3 ± 2.1 years, 75.7 ± 9.9 kg)	Incremental exercise to exhaustion, followed by sprint and endurance exercise after 48 h (acute effects) and 15 days of supplementation (prolonged effects) with low or high doses of combination of natural polyphenols luteolin and mangiferin, or placebo	NIRO-200NX (TOI)	Vastus lateralis, vastus medialis (Sf: NA)	During sprint exercise, mangiferin + luteolin supplementation facilitated muscle O_2 extraction. Compared to placebo, mangiferin + luteolin increased muscle O_2 extraction during post-exercise ischemia while improving sprint performance. Similar responses elicited by the two tested doses
Goulding et al. (2020) [56]	9	Recreationally active subjects (M: 23 ± 3 years, 77 ± 8 kg)	Critical power determined in normoxia and hyperoxia (FiO_2 50%) via 4 severe-intensity constant load cycling tests to exhaustion	OxiplexTS (aO_2Hb, aHHb)	Right vastus lateralis and rectus femoris (Sf: NA)	Increased muscle aO_2Hb in hyperoxia resulted in increased critical power
Griffin et al. (2018) [57]	12	Recreationally active subjects (M: 30 ± 6 years, 82.1 ± 10.7 kg)	Two 3-min all-out cycling tests separated by 7 days preceded by ischemic preconditioning or sham conditions	PortaMon[d] (TSI, ΔHHb)	Right vastus lateralis (Sf: NA)	No differences in TSI or HHb kinetics between conditions during the 3-min all-out test
Hopker et al. (2017) [58]	14	Well-trained cyclists (M: 30 ± 14 years, 66 ± 11 kg)	2-h steady-state cycling bout at 60% of maximal power output	PortaMon[d] (TSI, ΔO_2Hb, ΔHHb, ΔtHb, mVO_2, O_2Hb half-recovery time)	Right vastus lateralis (Sf: NA)	mVO_2 increased being higher after 90 and 120 min than 5 min. O_2Hb half-recovery time increased comparing pre and post 2-h cycling exercise albeit not significantly different
Horiuchi et al. (2022) [59]	10	Trained cyclists (M: 21 ± 1 years, 66.8 ± 8.7 kg)	Five sets of 30-s leg cycling with a maximal effort with a 4-min recovery of unloaded cycling between the sets in hypoxia (FiO_2 14%). Load during pedaling corresponded to 7.5% of individual's body weight at the 1st set, and it gradually reduced from 6.5 to 5.5, 4.5, and 3.5% for the 2nd to 5th sets	BOM-L1TRW (StO_2, ΔO_2Hb, ΔHHb, ΔtHb, StO_2 area under hyperemic curve)	Vastus lateralis (Sf: NA)	StO_2 area under hyperemic curve above the baseline values during hypoxic recovery and lower than during normoxic recovery
Hovorka et al. (2022) [60]	11	Junior National team cyclists (3F/8 M: 14.3 ± 1.6 years, 52.7 ± 12.1 kg)	6-min step increase in work rate to moderate intensity over a period of 15 months	PortaMon[d] (ΔHHb)	Right vastus lateralis (Sf: 5.2 ± 1.5 mm)	No significant HHb changes observed

Iannetta et al. (2017) [61]	15	Well-trained cyclists (11 M: 30.5 ± 8.4 year, 76.5 ± 8.4 kg; 4 F: 30.5 ± 5.9 year, 61.9 ± 4.4 kg)	Ramp incremental cycling tests (30 and 25 W/min for M and F) performed on 2 different days separated by 48 h	OxiplexTS (aHHb)	Vastus lateralis (Sf: NA)	aHHb breaking-point repeatable measure consistently occurring towards the end of a ramp incremental cycling test. Association between aHHb breaking point and respiratory compensation point
Inglis et al. (2020) [62]	8	Highly trained amateur cyclists (M: 39 ± 5 years, 78 ± 8 kg)	Ramp-incremental test to exhaustion to determine respiratory compensation point and HHb break point before, in the middle and after 7-month training and racing season	OxiplexTS (aHHb)	Vastus lateralis (Sf: NA)	At all measured time points, aHHb break point was not different
Kent et al. (2018) [63]	12	Endurance-trained cyclists (M: 27 ± 6 years, 75.4 ± 10.0 kg)	Two 60-min sub-maximal cycling trials at 60% of VO_2peak performed in hot environmental conditions	Moxy[d] (SmO_2)	Right vastus lateralis (Sf: NA)	No muscle oxygenation differences between beetroot juice supplementation and placebo
Jo et al. (2019) [64]	29	Recreationally active subjects (14 F/15 M; nitrate supplement group (n: 14; 6 F/8 M), 23.4 ± 2.0 years, 72.2 ± 13.6 kg; placebo group (n: 15; 8 F/7 M), 22.2 ± 2.5 years, 68.1 ± 13.3 kg)	8 km simulated cycling time trial before and after a 14-day supplementation period with either nitrate supplement or placebo	Moxy[d] (SmO_2)	Dominant leg vastus lateralis (Sf: NA)	No SmO_2 differences over time and across treatments
Mattioni et al. (2018) [65]	10	Recreationally trained cyclists (3 F/7 M: 25 ± 3 years, 70.3 ± 9.9 kg)	VO_2 kinetics during three step transitions with two priming 3- and 6-min exercise conditions and post-VO_2 kinetics test	OxiplexTS (aHHb)	Vastus lateralis (Sf: NA)	3- and 6-min priming-exercise modalities resulted in reduction in aHHb/VO_2 ratio.
McLay et al. (2017) [66]	14	Recreationally active subjects (7 old M: 74 ± 6 years, 77.7 ± 6.0 kg; 7 young M: 25 ± 3 years, 82.4 ± 6.4 kg)	3 endurance cycling exercise training sessions at 70% VO_{2peak} 24, 48, 72, and 120 h after a short-term exercise training	OxiplexTS (aHHb)	Vastus lateralis (Sf: NA)	Both groups showed significant decrease in the aHHb/VO_2 at 24, 48, and 72 h after training
Morgan et al. (2019) [67]	8	Trained cyclists (M: 19.7 ± 1.6 years, 75.0 ± 9.6 kg)	Two 15-km time-trials in the shortest time possible on a time trial bike following 7-day of placebo and Montmorency cherry polyphenols supplementation	PortaMon[d] (TSI)	Vastus lateralis (Sf: NA)	Vasoactive supplementation improved 15-km cycling performance and baseline muscle oxygenation; more pronounced increase in TSI after supplementation at lower exercise intensities

Nimmerichter et al. (2020) [68]	15	Elite young cyclists (2 F/13 M: 13.9 ± 1.7 years, 51.3 ± 12.4 kg)	Two 6-min step-transitions (20 W/min to moderate- and heavy-intensity cycling work rate)	PortaMon[d] (TSI, ΔHHb)	Right vastus lateralis (Sf: 4.6 ± 0.8 mm)	Excellent reliability in amplitude and kinetics of HHb and TSI for both intensities
O'Grady et al. (2021) [69]	20	Well-trained competitive cyclists (2 F/18 M: 38 ± 11 years, 72.4 ± 9.2 kg)	3 exercise trials of 9 randomized self-paced bicycle exercise bouts of either 1, 4, or 8 min at rating of perceived exertions of 9, 13, and 17 (6 – 20 scale)	PortaMon[d] (TSI, ΔO_2Hb, ΔHHb, ΔtHb)	Vastus lateralis (Sf: NA)	Increased rating of perceived exertions associated with higher ΔHHb, and lower TSI and ΔO_2Hb. Lower TSI and higher ΔHHb when exercise time increased
Oueslati et al. (2017) [70]	14	Trained young cyclists (M: 14, 16 ± 2 years, 63 ± 9 kg)	Incremental ramp and constant-load heavy exercise	PortaMon[d] (ΔO_2Hb, ΔHHb, ΔtHb)	Vastus lateralis (Sf< 15 mm)	VO_2 slow component correlated to ΔtHb slow component during constant-load exercise
Porter et al. (2020) [71]	14	Amateur cyclists (M: 14, 25.9 ± 7.4 years, 77.7 ± 11.0 kg)	10 x 15 s sprints with 45 s passive recovery on cycle ergometer	PortaMon[d] (TSI)	Right vastus lateralis (Sf: 10.5 ± 4.1 mm)	O_2 supplement (FiO_2 100%) improved muscle oxygenation and performance
Raberin et al. (2023) [72]	10	Recreationally active subjects (M: 29.2 ± 6.0 years, 76.5 ± 3.0 kg)	3 repeated sprint exercise to exhaustion with the same exercise-to-rest ratio (1:2), but different sprint durations (5, 10, or 20 s) in normoxic and hypoxic condition (FiO_2 13.6%).	OxyMon MkIII (ΔHHb, ΔtHb)	Vastus lateralis (Sf: NA)	Muscle deoxygenation impacted by sprint duration on ΔHHb and ΔtHb, but not by hypoxia
Raleigh et al. (2018) [73]	31	Competitive cyclists and triathletes (M: 29 ± 9 years, 77.7 ± 10.0 kg)	Graded incremental cycling (25 W/3-min) to volitional exhaustion	Moxy[d] (SmO_2)	Left vastus lateralis (Sf: NA)	SmO_2 threshold not a suitable non-invasive determinant of aerobic-anaerobic transition when compared to lactate invasive measure
Rasica et al. (2022) [74]	31	Trained and untrained subjects (15 trained: 8 M, 22 ± 2 years, 75.1 ± 4.2 kg; 7 F, 21 ± 1, 62.0 ± 7.9 kg. 16 untrained: 8 M, 24 ± 4 years, 71.5 ± 6.7 kg; 8 F, 23 ± 3 years, 56.7 ± 5.1 kg)	Cycling incremental exercise test to exhaustion	PortaMon[d] (TSI, ΔO_2Hb, ΔHHb, ΔtHb)	Right vastus lateralis (Sf_u: trained, 4.6 ± 1.0, 5.8 ± 1.6 mm; untrained, 5.4 ± 1.4, 4.1 ± 6.6 mm)	TSI decrease (during occlusion) and recovery rates faster in trained versus untrained, and M vs. F. VO_2max correlated with TSI reperfusion rate
Rokkedal-Lausch et al. (2019) [75]	12	Well-trained cyclists (M: 29.1 ± 7.7 years, weight NA)	10-km cycling time trial	OxyMon MkIII (ΔO_2Hb, ΔHHb, ΔtHb)	Right vastus lateralis (Sf: NA)	No effect of dietary nitrate supplementation via beetroot juice on muscle oxygenation during time trial

Rokkedal-Lausch et al. (2021) [76]	12	Well-trained cyclists (M: 29.1 ± 7.7 years, weight NA)	Three transitions from rest to moderate intensity cycling in hypoxia and normoxia with supplementation of beetroot juice or placebo	OxyMon MkIII (ΔO_2Hb, ΔHHb, ΔtHb)	Right vastus lateralis (Sf: NA)	Multiple-day, high-dose dietary nitrate supplementation via beetroot juice did not alter muscle oxygenation during normoxia and hypoxia
Rupp et al. (2022) [77]	13	Trained cyclists (M: 34.7 ± 9.5 years, 75.2 ± 7.2 kg)	Three cycling time-trials (250 kJ) after 24 h normobaric hypoxia at a simulated altitude of 3,450 m, hypobaric hypoxia at a terrestrial altitude of 3,450 m, and normobaric normoxia as control	OxyMon MkIII (ΔHbdiff, ΔtHb)	Vastus lateralis (Sf: NA)	Hbdiff lower in normobaric hypoxia compared to: i) control from 20 to 210 kJ, and lower in control compared to hypobaric hypoxia from 190 to 240 kJ; ii) hypobaric hypoxia from 50 kJ onward. No difference in HHb increase during time-trials between conditions
Saito et al. (2018) [78]	18	9 cycle-based athletes (7 triathletes and 2 cyclists; 2 F/7 M: 21.3 ± 1.5 years, 59.8 ± 7.7 kg); 9 nonathletic (2 F/7 M: 26.0 ± 4.7 years, 63.5 ± 11.0 kg)	Incremental cycling exercise at constant cadence of 90 rpm. Riding postures were: racing posture using an aero-handle bar (aero posture) and usual upright racing posture as the control	NIRO-200 (TOI)	Right vastus lateralis and rectus femoris (Sf: NA)	In cycle-based athletes, TOI changes of vastus lateralis and rectus femoris in aero posture lower than in upright posture throughout exercise. Difference between postures in TOI changes of rectus femoris in the final phase of exercise in nonathletic subjects
Salas-Montoro et al. (2022) [79]	90	Elite cyclists (58 F: 21.4 ± 5.5 years, 56.8 ± 6.2 kg; 32 M: 21.7 ± 6.2 years, 67.6 ± 4.8 kg)	Ramp incremental test on cycle ergometer (25 W/min)	Humon Hex[d] (SmO_2)	Right rectus femoris (Sf: NA)	No differences between SmO_2 breakpoint and 2nd lactate threshold. No differences between M and F
Shastri et al. (2019) [80]	12	Moderately trained subjects (M: 29 ± 10 years, 74 ± 11 kg)	Two sub-GET 4-min cycling tests pedaling at 40, 50, 60, 70, 80 and 90 rpm cadence (1st at 70% and 2nd at 90% GET)	PortaMon[d] (TSI, ΔO_2Hb, ΔHHb)	Right vastus lateralis (Sf: 8.0 ± 4.8 mm)	TSI decreased from rest to exercise when pedaling at 90 rpm, compared to pedaling at 40 and 50 rpm
Shaw et al. (2020) [81]	12	Trained cyclists (2 F/ 10 M: 35 ± 12 years, 75.2 ± 11.0 kg)	After 2 weeks of dark chocolate supplementation (120 g/day), 90 min at 50% peak power output, followed immediately by 10-km time trial at simulated altitude (FiO_2 15%)	PortaMon[d] (ΔO_2Hb, ΔHHb, ΔtHb)	Vastus lateralis (Sf: NA)	Increase in O_2 extraction during prolonged exercise; no effects of supplementation in vastus lateralis oxygenation
Skovereng et al. (2017) [82]	13	Recreationally trained cyclists (M: 40.5 ± 5.0 years, 81.7 ± 7.4 kg)	Constant load cycling at different cadences (from 60 to 110 rpm)	PortaMon[d] (TSI, ΔHHb, mVO_2)	Gastrocnemius, tibialis anterior (Sf: NA)	Increasing cadence increased deoxygenation for both muscle groups. Higher post exercise mVO_2 following increased cadence above 80 rpm only in tibialis anterior

Stöcker et al. (2017) [83]	17	Recreationally active subjects (M: 28 ± 5 years, 77 ± 9 kg)	3 min cycling bouts at 6 different intensities (40–90% peak VO_2, separated by 5 min rests)	PortaMon[d] (ΔO_2Hb, ΔHHb, ΔtHb)	Vastus lateralis (Sf: 7.5 ± 3.1 mm)	$\Delta HHb/\Delta VO_2$ increased from 40% to 60% VO_{2peak} and decreased from 60% to 90% VO_{2peak}. Overshoot in post-exercise ΔtHb and ΔO_2Hb related to pre-exercise values (equal after 40–60% VO_2peak and higher thereafter)
van der Zwaard et al. (2018) [84]	28	6 international sprint, 8 team pursuit,14 road cyclists (M: 25 ± 7 years, 77.4 ± 8.0 kg)	Wingate test (sprint) and 15-km time trial (endurance)	PortaMon[d] (ΔO_2Hb, ΔHHb)	Vastus lateralis (Sf: NA)	Performance VO_2 and O_2 supply capacity (mean corpuscular hemoglobin concentration and ΔO_2Hb) explained 92% of variance in normalized endurance performance
Wakabayashi et al. (2018) [85]	8	Physically active subjects (M: 71.7 ± 12.4 kg)	After lower body immersion (12°C water for 30 min), 30-min cycling exercise at lactate threshold with thigh cooling by a water-circulating pad. Controls, no cooling	BOM-L1TRW (StO_2, ΔO_2Hb, ΔHHb, ΔtHb)	Vastus lateralis (Sf: NA)	After exercise onset, O_2Hb % change lower in cooling compared with controls. After exercise onset, HHb % change showed a transient peak and higher value in cooling
Willis et al. (2017) [86]	11	Recreationally active subjects (5 F/6 M: 26.7 ± 4.2 years, 68.0 ± 14.0 kg)	Two 10-sprints and repeated cycling sprints (10-s sprint: 20-s recovery) test to exhaustion in 3 conditions of simulated altitude of 380, 2,000 and 3,800 m (FiO_2 21, 16 and 13 %)	PortaMon[d] (TSI, ΔO_2Hb, ΔHHb, ΔtHb, ΔHbdiff)	Vastus lateralis (Sf: NA)	TSI and Hbdiff lower at 3,800 than at 2,000 and 400 m, and lower HHb at 3,800 compared with 2,000 m. Reduced changes in TSI, Hbdiff and tHb throughout the test to exhaustion
Willis et al. (2018) [87]	11	Physically active subjects (5 F/6 M: 26.7 ± 4.2 years, 68.0 ± 14.0 kg)	Repeated cycling sprint test to exhaustion (10-s maximal sprints with 20-s recovery until exhaustion) with proximal lower limb bilateral vascular occlusion at 0, 45, and 60% of resting pulse elimination pressure	PortaMon[d] (TSI, ΔtHb, ΔO_2Hb, ΔHHb)	Vastus lateralis (Sf: NA)	ΔHHb decrease during sprints at 60% compared to 0%; ΔtHb increase at both 45 and 60% compared with 0%
Woorons et al. (2017) [88]	9	4 well trained cyclists, 5 team players (4 basketball and 1 rugby) (F:1 cyclist; M: 27.2 ± 9.3 years, 74.7 ± 9.6 kg)	Two sets of 8x 6-s sprints on a cycle ergometer followed by 24 s inactive recovery carried out with normal breathing or voluntary hypoventilation at low lung volume	PortaMon[d] (ΔHHb, ΔtHb)	Left vastus lateralis (Sf: NA)	Changes in HHb and tHb greater in voluntary hypoventilation

	Woorons et al. (2019) [89]	18	Competitive cyclists (M: 34.6 ± 11 years, 73.2 ± 9 kg)	Six repeated-sprint sessions in cycling either with hypoventilation at low lung volume or with normal conditions. Before and after training period, subjects performed 10 × 6-s all-out cycling sprints	PortaMon[d] (ΔHHb, ΔtHb)	Right vastus lateralis (Sf < 7 mm)	Similar HHb increase in both groups accompanied by no tHb changes
	Yamaguchi et al. (2021) [90]	11	Endurance and resistance-trained athletes (M: 19.4 ± 0.4 years, 72.8 ± 2.0 kg)	3 sets of 5 × 6-s maximal cycling sprints with 30-s active recovery under control (23 °C, 21 % FiO_2, hypoxic (23 °C, 15 % FiO_2), or hot + hypoxic (35 °C, 15% FiO_2) conditions	Hb14 (StO_2, mBF)	Right vastus lateralis (Sf < 7 mm)	End-exercise StO_2 lower in hypoxia than in normoxia, but no difference between hot/hypoxic condition and normoxia. Hot/hypoxic condition associated with higher mBF compared with control and hypoxia
	Yogev et al. (2022) [91]	22	Trained cyclists (5 F/17 M: 31 ± 8 years, 75 ± 12 kg)	Ramp incremental test on cycle ergometer to exhaustion (30 W/min)	Moxy[d] (SmO_2)	Right and left vastus lateralis, right lateral deltoid (Sf: NA)	No difference in locomotor or non-locomotor muscle deoxygenation breakpoint
	Zurbuchen et al. (2020) [92]	22	11 trained cyclists (M: 27.4 ± 5.1 years, 72.3 ± 2.8 kg), 11 active subjects (M: 28.7 ± 5.1 years, 77.7 ± 8.1 kg)	Submaximal incremental cycling test with workload increased by 7.5 % maximal aerobic power for active subjects and 10% for cyclists every 5 min until the end of the stage with respiratory exchange ratio of 1	OxyMon MkIII (ΔHHb, ΔtHb)	Right vastus lateralis (Sf: NA)	Quadriceps deoxygenation kinetics related to whole body fat oxidation. Greater muscle capacity to use O_2 to oxidize fat during incremental exercise in endurance trained cyclists
**Diving**	Costalat et al. (2017) [93]	16	Trained breath-hold divers vs. non-divers (M: 8 divers, 38.8 ± 8.6 years, 79.5 ± 15.0 kg; 8 controls, 36.0 ± 7.8 years, 77.5 ± 15.5 kg)	Simulated dynamic breath-holding with cold face immersion while steadily pedaling at 30% of peak power output	PortaMon[d] (TSI, ΔO_2Hb, ΔHHb, ΔtHb)	Vastus lateralis (Sf: NA)	Lower HHb increase for trained breath-hold divers vs. controls at cessation of breath holding when tHb decreased only in trained divers
	Myers et al. (2018) [94]	15	Well-trained divers (15 M: 29.3 ± 1.2 years, 88.2 ± 10.3 kg)	5 consecutive days 6-h resting water immersions breathing atmospheric air at 1.35 atmospheres. 40 % maximal handgrip endurance and 50-repetition maximal isokinetic knee extensions before and after water immersions	PortaMon[d] (TSI, ΔHHb)	Right vastus lateralis, flexor carpi radialis (Sf < 17 mm; NA)	No change in oxygenation kinetics in load bearing and non-load bearing muscles

	Myers et al (2019) [95]	13	Well-trained divers (13 M: 31.3 ± 1.7 years, 81.4 ± 2.8 kg)	5 consecutive days 6-h resting water immersions breathing 100 % O_2 at 1.35 atmospheres. 40 % maximal handgrip endurance and 50-repetition maximal isokinetic knee extensions before and after water immersions	PortaMon[d] (TSI, ΔO_2Hb, ΔHHb, ΔtHb)	Right vastus lateralis, flexor carpi radialis (Sf < 17 mm NA)	No significant limitations in O_2 offloading kinetics during exercise and muscle oxidative capacity measurements. No effect of hyperoxia on O_2 offloading kinetics or mitochondrial function
**Football**	Abaïdia et al. (2019) [96]	10	Trained football player (10 M: 26.1 ± 4.9 years, 77.2 ± 9.1 kg)	Single-leg knee flexor exercise: 5 sets of 15 maximal eccentric contractions on isokinetic dynamometer. 20 h after exercise, 12 min intermittent cycling exercise (15 s work/15 s rest) or control. Two weeks after, same protocol on other leg	PortaMon[d] (ΔO_2Hb, ΔHHb)	Biceps femoris (Sf: NA)	Increases in hamstring oxygenation using intermittent cycling the day after exercise-induced muscle damage as compared to passive recovery
	Archiza et al. (2018) [97]	18	Professional football players (F: 8 controls, 20.1 ± 2.0 years, 56.4 ± 5.8 kg; 10 trained, 22.0 ± 3.9 years, 57.1 ± 7.4 kg)	Pre- and post-6-week inspiratory muscles training, high-intensity constant speed treadmill exercise to exhaustion (100% of speed reached during maximal incremental test), and repeated sprint ability test: six 40 m (20 m + 180º turn + 20 m) sprints on a grass field	OxyMon MkIII (ΔO_2Hb, ΔHHb, ΔtHb)	Intercostal of left 7th intercostal space, vastus lateralis (Sf: NA)	Greater decrease of HHb and tHb on intercostal muscle concomitantly to greater increase of O_2Hb and tHb on vastus lateralis during high-intensity exercise to exhaustion after 6 weeks training
	Bonilla et al. (2020) [98]	14	Spain 2nd division football players (F: 27.8 ± 5.0 years, 71.6 ± 3.3 kg)	mVO_2 measurement before, immediately after and 24 h after match	Moxy[d] (SmO_2, mVO_2)	Gastrocnemius (Sf: NA)	Increase in SmO_2 kinetics, and resting mVO_2 24 h after match
	Bujalance-Moreno et al. (2020) [99]	16	Amateur level football players (M: 23.9 ± 4.2 years)	Four 4-min bouts played on a pitch size of 30 x 20 m, with a 2-min passive recovery between bouts	Moxy[d] (SmO_2, tHb)	Dominant leg vastus lateralis (Sf: NA)	No differences between bouts or recovery periods for SmO_2 and tHb changes
	Caruso et al. (2020) [100]	10	Professional football players (F: 23 ± 4 years, 53 ± 5 kg)	Constant-load tests on a treadmill until exhaustion	OxyMon MkIII (TSI, ΔO_2Hb, ΔHHb, ΔtHb)	Intercostal of left 7th intercostal space, vastus lateralis (Sf: NA)	High intensity exercise with inspiratory loading decreased peripheral muscle oxygenation more than sham condition with negative impact on exercise performance
	Michailidis et al. (2020) [101]	10	Young football players (M: 16.6 ± 0.9 years, 64.7 ± 6.6 kg)	Repeated anaerobic sprint test involving 6 × 35-m straight line maximal sprints in alternating	Moxy[d] (SmO_2, T1/2 SmO_2)	Right vastus lateralis (Sf < 15 mm)	Muscle reoxygenation rate inversely correlated (r = -0.71) with aerobic capacity (VO_2max)

				directions interspersed by 10 s recovery in synthetic grass field			
	Vasquez-Bonilla et al. (2021) [102]	17	Football players competing in Spanish 3rd division (M: 22 ± 2 years, 74.5 ± 10.1 kg)	SmO_2 (before and during arterial occlusion) before and after preseason	Moxy[d] (SmO_2, mVO_2)	Bilateral gastrocnemius (Sf: NA)	Inverse relationship between SmO_2 at the initial-occlusion (r = -0.82), final-occlusion (r = -0.79) and SmO_2 recovery (r = -0.82) with the single leg counter movement jump power
	Vasquez-Bonilla et al. (2021) [103]	38	Professional football player (F: 22.5 ± 3.8 years, 60.7 ± 6.6 kg)	8 sprints x 20 m, 20 s recovery	Moxy[d] (SmO_2)	Gastrocnemius medialis (Sf: 4.2 ± 1.3 mm)	Progressive SmO_2 increase during repeated sprint ability test
	Vasquez-Bonilla et al. (2022) [104]	25	Spanish III Division football (M: 25, 22.4 ± 2.6 years, 74.8 ± 9.9 kg)	8 sprints x 20 m, 20 s recovery	Moxy[d] (SmO_2)	Gastrocnemius medialis (Sf: 9.3 ± 2.3 mm)	SmO_2 changes as the most sensitive variable to detect the minimal change in repeated-sprint ability performance
**Futsal**	dos Santos et al. (2020) [105]	13	Amateur futsal players (F: 24.1 ± 3.7, 63.6 ± 8 kg)	Testing battery: counter.movement jump, Illinois agility, YoYo intermittent recovery test level 1 preceded by 15 min of photobiomodulation therapy	Moxy[d] (SmO_2, tHb)	Right vastus lateralis (Sf: NA)	SmO_2 changes during YoYo intermittent recovery test did not differ between treatment and placebo as did intermittent performance
**Kayak**	Matzka et al. (2021) [106]	14	Elite sprint kayakers (6 F: 17.8 ± 2.5 years, 65.8 ± 4.8 kg; M 8: 19.4 ± 4.4 years, 83.3 ± 10.3 kg)	5 × 1500-m incremental kayak test at different intensities (60, 70, 80, and 90% of maximal heart rate, as well as an all-out effort) performed on an ergometer and on-water	PortaMon[d] (TSI)	Right biceps brachii (Sf: NA)	TSI during the all-out incremental phase 21% lower during on-water than ergometer kayaking; TSI correlated with 1000-m race performance, (r = 0.62)
	Paulauskas et al. (2022) [107]	1	World-class kayaker (M: 1, 32 years, 89 kg)	Repetitive training (6 bouts at 200 W of 6 min, 6 min recovery), interval training (6 bouts of 6 min at 200 W, 1 min relief at 40 W) and sprint interval training (6 bouts of 6 min with interspersing periods of 10 s at 300 W) protocols on a kayak ergometer	Moxy[d] (SmO_2, tHb)	Left latissimus dorsi, pectoralis major, vastus lateralis (Sf< 25 mm)	Different SmO_2 and tHb changes in three muscle groups in response to different training stimuli
	Yamaguchi et al. (2019) [108]	8	Trained kayakers (M: 19.8 ± 0.4 years, 71.1 ± 2.2 kg)	Repeated double-poling sprint (3 × 3 × 20-s maximal sprints, 40-s passive recovery, 5-min rest) in either hypoxia (FiO_2 14.5%) or normoxia	Hb14 (ΔO_2Hb, ΔHHb, Δt Hb)	Triceps brachii (Sf: NA)	Greater HHb and tHb increases in hypoxia

**Lacrosse**	Kojima et al. (2021) [109]	7	Lacrosse players (7 M: 21.7 ± 0.8 years, 65.9 ± 4.7 kg)	A 1000 m all-out row testing and four 30-s sprints (in 6 oarsmen) on a wind-braked ergometer	Hb14 (StO_2, ΔO_2Hb, ΔHHb, ΔtHb)	Right vastus lateralis (Sf: NA)	mBF restriction during rest periods decreased StO_2 and O_2Hb without interfering with power output during sprints
**Long-track speed skating**	Piucco et al. (2018) [110]	12	Well-trained young long-track speed skaters (8 F/4 M: 18.0 ± 0.9 years, 65.0 ± 6.8 kg)	Maximal incremental test skating on a treadmill or skating on a slide board	Moxy[d] (HHb)	Right vastus lateralis (Sf: NA)	Similar muscle deoxygenation kinetics between slide-board and treadmill skating
	Richard and Billaut (2018) [111]	9	Elite long-track speed skaters (2 F/7 M: 23.3 ± 2.6 years, 78.8 ± 10.3 kg)	1000-m on ice time trial preceded by three 5-min ischemic preconditioning of upper limbs	PortaMon[d] (TSI, ΔO_2Hb, ΔHHb, ΔtHb)	Vastus lateralis (Sf: 16.6 ± 4.5 mm)	No improvement in skating performance and muscle oxygenation after ischemic preconditioning
	Richard and Billaut et al. (2018) [112]	7	Elite long-track speed skaters (M: 7, 23.4 ± 3.3 years, 80.9 ± 7.7 kg)	Two 600-m race simulations on an indoor long-track (400-m) speed-skating oval, preceded by either a combination of preconditioning strategies (upper limbs ischemic preconditioning (3 × 5-min at 180 mmHg) over 3 days with addition of an inspiratory muscle warm-up or placebo	PortaMon[d] (TSI, ΔO_2Hb, ΔHHb, ΔtHb)	Right vastus lateralis (Sf: 7.7 ± 2.5 mm)	Mean and peak HHb changes respectively likely and possibly higher in the last section of preconditioning race (presumably a higher O_2 extraction) with no performance-enhancing effect
	Richard and Billaut et al. (2019) [113]	8	Elite long-track speed skaters (3 F/ 5M: 21.4 ± 3.5 years, 75.4 ± 11.2 kg)	3000 m on-ice time trials, preceded by either inspiratory muscle warm-up (2 × 30 breaths, 40% maximal inspiratory pressure) or control	PortaMon[d] (TSI, ΔO_2Hb, ΔHHb, $\Delta Hbdiff$, ΔtHb)	Right vastus lateralis (Sf: 8.7 ± 3.3 mm)	No effect of inspiratory muscle warm-up on TSI, Hbdiff, tHb, O_2Hb, and skating time performance
**Marathon**	Contreras-Briceño et al. (2019) [114]	15	Marathon runners (M: 24.9 ± 2.0 years, 65.2 ± 6.2 kg)	Incremental maximal treadmill exercise until exhaustion (increase of 2 km/h every 150 s)	Moxy[d] (SmO_2)	Vastus lateralis, intercostal of 7th intercostal space (Sf: NA)	SmO_2 reliability for intercostal good at low intensity and excellent at higher intensity. Intercostal SmO_2 inversely related with minute ventilation, respiratory rate, and tidal volume
	Contreras-Briceño et al. (2021) [115]	19	High-level competitive marathon runners (M: 22.9 ± 1.9 years, 66.5 ± 6.7 kg)	Incremental treadmill exercise until voluntary exhaustion (increase of 2 km/h every 150 s, slope 2%)	Moxy[d] (SmO_2, tHb)	Right vastus lateralis, intercostal of right 7th intercostal space (Sf: NA)	Deoxygenation of the vastus lateralis more important than the intercostal muscle from the 1st GET. Deoxygenation of intercostales during incremental maximal exercise associated with the respiratory rate. Correlation between maximum changes of SmO_2 in all muscles during exercise with VO_2peak

	Jones et al. (2017) [116]	27	Physically active subjects (10 F/7 M: 29.4 ± 3.6 years, 72 ± 13 kg)	mVO_2 at rest and immediately following a maximal exercise test at ~6 months prior to the subject's 1st marathon, and within 3 weeks after marathon	PortaMon[d] (ΔO_2Hb, ΔHHb, mVO_2)	Lateral gastrocnemius (Sf_u: 6.0 ± 2.2 mm)	Post-training associated with 48% increase in post-exercise mVO_2
	Kerhervé et al. (2017) [117]	14	Trained runners (M: 21.7 ± 3.0 years, 72.3 ± 6.7 kg)	Two 24-km trail running sessions wearing degressive calf compression (23 mmHg) sleeves or control (<4 mmHg) sleeves	PortaMon[d] (TSI, ΔO_2Hb, ΔHHb, ΔtHb, mBF, mVO_2)	Right gastrocnemius medialis (Sf: NA)	Improved muscle oxygenation with compression before and after exercise (stationary position) without affecting muscle oxygenation during exercise
**Nordic walking**	Paredes-Ruiz et al. (2020) [118]	30	Recreational Nordic Walking athletes (18 M: 50.1 ± 6.6 years, 81.1± 9.0 kg; 12 F: 53.7 ± 2.2 years, 67.3 ± 7.2 kg)	Maximal exercise testing in treadmill according to a modified Bruce protocol on a ramp	Humon Hex[d] (SmO_2)	Dominant leg vastus lateralis (Sf: NA)	Continuous SmO_2 decrease with the exercise intensity until exhaustion. Minimum SmO_2 at VO_2max
**Resistance-training**	Guardado et al. (2021) [119]	12	2 years continuous resistance-training experience (4 F/8 M: 27.1 ± 5.7 years, 72.3 ± 13.4 kg)	Barbell bench press during 3 different training sessions	Moxy[d] (SmO_2)	Intercostal of 4th intercostal space	No SmO_2 difference between 3 resistance-training sessions under different training protocols focused on upper body exercises
	Halley et al. (2019) [120]	11	Trained athletes (M: 23.1 ± 2 years, 78.6 ± 7 kg)	Four exercise trials: 6 sets of 11 repetitions of maximal effort dynamic single-leg extensions (120°/s extension; 300°/s flexion; 40 reps/min) in either normoxic or hypoxic (FiO_2 14%) conditions, preceded by either ischemic preconditioning (three 5-min bilateral leg occlusions at 220 mmHg) or sham. Each set lasted ~17 s with 20 s of rest between each set	MoorVMS-NIRS (SO_2, ΔO_2Hb, Δ HHb, ΔtHb)	Rectus femoralis (Sf: mean value 14.7 mm)	Reduced SO_2 in both hypoxic conditions compared with normoxia with an even further reduction of 3% in hypoxic ischemic preconditioning compared with sham. Improved muscle O_2 extraction during exercise with FiO_2 reduction, but without performance benefit
	Haynes JT 4th et al. (2021) [121]	10	Resistance-trained athletes (10 M: 22.6 ± 3.2 years, 88.3 ± 7.8 kg)	mVO_2 at rest and immediately following a maximal exercise test at ~6 months prior to the subject's 1st marathon, and within 3 weeks after marathon	Moxy[d] (SmO_2)	Anterior deltoid (Sf: NA)	7 days of red spinach extract supplementation did not change SmO_2 dynamics during or following bench press exercise, as well as performance
	Marshal et al. (2020) [122]	16	Resistance-trained subjects (8 F: 25.6 ± 5.9 years, 71.0 ± 8.6 kg; 8 M:	Two sets of 10 isokinetic contractions (60°/s) with knee extensors	PortaMon[d] (TSI, ΔHHb)	Right rectus femoris (Sf < 20 mm)	Greater muscle oxygenation for women during the isokinetic repetitions with no influence on neuromuscular fatigue features

			25.5 ± 6.2 years, 86.4 ± 9.8 kg)	performed before and after exercise session (full-body resistance exercise based on real-world athletic practice), as well as 1, 24, and 48 h after session			
	Scott et al. (2017) [123]	12	Resistance-training subjects (M: 21-29 years, 84.6 ± 11.6 kg)	Moderate-load resistance exercise in normoxia and moderate-level hypoxia (FiO_2 16%) (3 sets of 10 repetitions of squats and deadlifts at 60% of 1 repetition maximum, with 60-s inter-set rest)	PortaMon[d] (ΔO_2Hb, ΔHHb)	Right vastus lateralis (Sf: NA)	No muscle oxygenation changes to moderate-load resistance exercise between conditions
**Roller ski skating**	Seeberg et al. (2021) [124]	9	Elite cross-country skiers (M: 25.9 ± 2.2, 80.1 ± 5.5)	Two 21-min treadmill roller skiing sessions (7 × 3 min laps) at low- and high-intensity with the same set inclines and intensity-dependent speeds	PortaMon[d] (TSI)	Right triceps brachii, vastus lateralis (Sf: 6.1 ± 1.6, 7.5 ± 1.5 mm)	Decreases in TSI for both arms and legs during low- and high-intensity sessions. Higher TSI decrease in vastus lateralis than triceps brachii between training intensities. Difference in terrain-dependent TSI fluctuations between arms and legs without being associated with pole versus ski power generated by arms versus legs, respectively
**Rowing**	Klusiewicz et al. (2021) [125]	8	Senior elite rowers (M: 29.2 ± 4.7 years, 89.5 ± 10.0 kg)	Incremental progressive submaximal exercise test and a 30-min test (84 % of anaerobic threshold) on rowing ergometer	Moxy[d] (SmO_2, tHb)	Right vastus lateralis (Sf: NA)	SmO_2 reduction (2.9%) between the 10th and 30th min of exercise with no tHb changes
	Possamai et al. (2022) [126]	11	Trained rowers (M: 20 ± 3 years, 78.4 ± 4.8 kg)	Isometric knee extension performed until HHb reached a target of ~50% of the physiological range amplitude followed by 12 brief arterial occlusions (six 3-s occlusions with 12 s recovery; three 6-s occlusions with 30 recovery; three 6-s occlusions with 60 s recovery	PortaMon[d] (TSI, ΔO_2Hb, ΔHHb, ΔtHb, mVO_2)	Vastus lateralis (Sf: 13.0 ± 6.0 mm)	mVO_2 recovery constant rate strongly associated with relative VO_2max (and peak power output), and unrelated with 2000-m rowing performance
	Turnes et al. (2018) [127]	16	Trained rowers (M: 24 ± 11 years, 74.1 ± 5.9 kg)	2000-m rowing ergometer test preceded by intermittent bilateral cuff inflation of lower limbs with three 5/10-min	PortaMon[d] (TSI, ΔO_2Hb, ΔHHb)	Vastus lateralis (Sf: NA)	10-min ischemia–reperfusion reduced TOI and O_2Hb during exercise compared with control without improvement of the 2000 m rowing performance

				ischemia–reperfusion cycles or control			
**Rugby**	Vasquez-Bonilla et al. (2023) [128]	22	1st division rugby players (M: 22.5 ± 4.6 years, 89.8 ± 12.6 kg)	High-intensity isokinetic fatigue on a dynamometer seat consisting of 30 consecutive maximal concentric contractions at an angular speed of 180°/s	Moxy[d] (SmO_2)	Bilateral vastus lateralis (Sf: NA)	Bilateral asymmetry in minimum SmO_2 and critical oxygenation between dominant and non-dominant vastus lateralis. Greater muscle mass associated with a better O_2 extraction ($r = 0.53$)
**Rugby 7**	Pramkratok et al. (2021) [129]	14	Professional rugby seven players (7 M: 24.1 ± 4.4 years, 80.4 ± 5.7 kg); controls (7M: 23.4 ± 4.9 years, 78.1 ± 15.3 kg)	Incremental running test until volitional exhaustion	PortaMon[d] (ΔTSI, ΔO_2Hb, ΔHHb, ΔtHb)	Left vastus lateralis (Sf: NA)	Enhanced vastus lateralis ΔO_2Hb and ΔtHb during maximal exercise in acute hypoxia (FiO_2 14.5 %) versus normoxia
	Pramkratok et al. (2022) [130]	14	Professional rugby seven players (Hypoxia: 7 M: 24.1 ± 4.4 years, 80.4 ± 5.7 kg; Normoxia: 7 M: 23.4 ± 4.9 years, 78.1 ± 15.3 kg)	Incremental running test until volitional exhaustion	PortaMon[d] (ΔTSI, ΔO_2Hb, ΔHHb, ΔtHb)	Left vastus lateralis (Sf: NA)	Enhanced vastus lateralis ΔO_2Hb and ΔtHb by adding a 6-week repeated sprint training in hypoxia (FiO_2 14.5 %) versus normoxia to usual training
**Running**	Balsalobre-Fernández et al. (2018) [131]	12	Elite middle and long-distance runners (M: 26.3 ± 5.1 years, 69.2 ± 8.6 kg)	Incremental running test to exhaustion on treadmill before and after 15-days supplementation period, in which half group consumed daily nitrate-rich beetroot juice (vs. placebo)	Moxy[d] (SmO_2)	Dominant leg vastus lateralis of (Sf: NA)	Higher vastus lateralis SmO_2 during exercise in group with nitrate-rich beetroot supplementation
	Bellinger et al. (2020) [132]	24	Highly trained middle-distance runners competitive middle-distance (800 and 1500 m) runners (16 M: 21.0 ± 3.6 years, 70.6 ± 7.9 kg; 8 F: 21.3 ± 3.2 years, 53.1 ± 6.0 kg)	3-weeks normal training, 3-weeks high-volume training, 1-week exponential reduction in training. Before and after each training period, gastrocnemius rate of mVO_2 recovery measurement	OxyMon MkIII (ΔO_2Hb, ΔHHb, ΔtHb, mVO_2)	Gastrocnemius medialis (Sf: NA)	Muscle oxidative capacity increased in response to high-volume training only in runners ($n = 12$) that had substantially larger performance improvements after a taper period
	Born et al. (2017) [133]	17	Competitive runners (4 F: 30 ± 8 years, 59.9 ± 4.8 kg; 13 M: 29 ± 4 years, 71.9 ± 5.6)	Time trial on off-road trail course made up of two 7 km-laps including 6 steep uphill and downhill sections with an elevation gain of 486 m	PortaMon[d] (TSI)	Right vastus lateralis (Sf: NA)	TSI was inversely correlated to VO_2, and lower during uphill compared to downhill sections
	de Aguiar et al. (2022) [134]	13	Trained subjects (M: 21 ± 4 years, 77 ± 17 kg)	Three sets of 2 repetitions of 6-min running exercise at 90% 1st	PortaMon[d] (TSI, ΔO_2Hb, ΔHHb, ΔtHb, mVO_2)	Left gastrocnemius (Sf: 5.2 ± 1.3 mm)	Reliable mVO_2 kinetics following moderate running exercise (good to

			GET. 18 intermittent arterial occlusions performed immediately after each running bout			excellent). Correlation between mVO_2 kinetics and parameters of aerobic fitness (maximal speed, GET and pulmonary VO_2 on-kinetics)
Fleming et al. (2017) [135]	25	Trained collegiate level distance runners (25 M: 23 ± 6 years, 69.9 ± 8.8 kg)	Self-paced 5000 m treadmill time-trial	PortaMon[d] (TSI)	Right rectus femoris (Sf: NA)	Decrease of TSI during exercise not affected by pre-exercise ingestion of a drink with higher dissolved O_2
Gagnon et al. (2017) [136]	11	Active runners (M: 24 ± 1 years, 80.0 ± 3.3 kg)	60-min treadmill sustained walking (at 50% VO_2peak) and running (at 70% VO_2peak) at 22°C and 0°C for skin cooling, and 0°C following core and skin cooling	OxyMon MkIII (ΔO_2Hb, ΔHHb, ΔtHb, ΔHbdiff)	Vastus lateralis (Sf: NA)	Greater deoxygenation changes during early stages of exercise with core tissue cooling, irrespective of tHb changes. No influence of skin cooling alone on Hbdiff during exercise
Hobbins et al. (2019) [137]	19	Trained runners (3 F/16 M: 33.4 ± 9.1 years, 76.3 ± 10.9 kg)	High-intensity interval treadmill running (4 x 4 min intervals) in normoxia and hypoxia (FiO_2 15%)	PortaLite (ΔO_2Hb, ΔHHb, ΔtHb)	Right vastus lateralis (Sf: NA)	O_2Hb, tHb decreased and HHb increased from interval 1-4 independent of condition
Jeffries et al. (2018) [138]	20	Endurance running or team sports athletes (M: 26 ± 5 years, 80 ± 12 kg)	Effects of 7 days of bilateral lower limb ischemic preconditioning (4 × 5 min; *n* = 10), or sham on: 1) mVO_2 (arterial occlusions at rest) or in a series of 15 brief occlusions after 15-s electrical stimulation, and 2) mBF during recovery from submaximal isometric plantar flexion exercises at 40 and 60% MVC	PortaMon[d] (ΔO_2Hb, ΔHHb, ΔtHb, mBF, mVO_2)	Gastrocnemius medialis (Sf: 6.9 ± 2.6 mm intervention group; 6.4 ± 2.5 mm sham)	Following intervention period, beyond the late phase of protection (72 h), muscle oxidative recovery kinetics speeded by 13%, and resting mVO_2 reduced by 16.4%. During exercise, HHb changes from rest to steady state reduced at 40 and 60% MVC (16 and 12%, respectively) despite similar tHb. At exercise end, the time constant for O_2Hb recovery accelerated at 40 and 60% MVC (by 33 and 43%, respectively)
Manchado-Gobatto et al. (2020) [139]	12	Physically active subjects (M: 22 ± 1 years, 71.4 ± 2.7 kg)	All-out 30-s tethered running on non-motorized treadmill and 18-min recovery	PortaMon[d] (TSI, ΔO_2Hb, ΔHHb, ΔtHb)	Right vastus lateralis, medial biceps brachii (Sf: NA)	TSI rapid adjustments (both, during and after running) in a tissue-dependence manner, with very low biceps brachii TSI during exercise when compared to vastus lateralis. Correlation between blood lactate and biceps brachii TSI
Marostegan et al. (2022) [140]	16	Physically active subjects (M:23 ± 1 years, 73.2 ± 2.0 kg)	Four trials 30 s all-out run on a non-motorized treadmill, preceded by shorter inspiratory	PortaMon[d] (TSI, ΔO_2Hb, ΔHHb, ΔtHb)	Right vastus lateralis and medial biceps brachii (Sf:	No changes in muscle oxygenation time course during the trials by any interventions. 40% of maximal

			muscle warm-up protocol (2 × 15 breaths with a 1-min rest interval, accomplished 2 min before 30 all-out run). Inspiratory muscle warm-up load conditions: 15, 40, and 60% of maximal inspiratory pressure		3.3 ± 0.2, 11.2 ± 1.2 mm)	inspiratory pressure improved biceps brachii TSI during passive recovery
Meirelles et al. (2019) [141]	11	Physically active subjects (M: 25 ± 2 years, 75.4 ± 9.5 kg)	Two 5-min treadmill runs (moderate, 90% of GET; heavy, 50% of the difference between GET and VO_2peak) 60 min after L-arginine or placebo supplementation	PortaMon[d] (ΔO_2Hb, ΔHHb, ΔtHb, ΔHbdiff)	Right vastus lateralis (Sf < 25 mm)	No muscle oxygenation differences between arginine and placebo conditions
Osawa et al. (2017) [142]	9	Distance runners and triathletes (M: 24 ± 3 years, 57.1 ± 3.6 kg)	Incremental running tests to exhaustion under normoxic and hypoxic conditions (FiO_2 15%)	NIRO-200NX (TOI, ΔO_2Hb, ΔHHb, ΔtHb)	Vastus lateralis, medial gastrocnemius (Sf: NA)	Both muscles TOI decreased under hypoxic compared with normoxic conditions at all running speeds. Greater vastus lateralis TOI decrease rate in hypoxia as running speed increased, with little change in medial gastrocnemius
Pawlack-Chaouch et al. (2019) [143]	20	10 trained runners and triathlon athletes (high fit, M: 25.6 ± 7.0 years, 67.9 ± 9.7 kg); 10 low exercise capacity group (low fit, M: 27.9 ± 6.2 years, 77.2 ± 7.9 kg)	Graded cycling exercise until volitional exhaustion	OxyMon MkIII (ΔHHb)	Left vastus lateralis (Sf: NA)	No difference in muscle oxygenation patterns during exercise between two groups. No association between plasma asymmetric dimethylarginine concentrations (endogenous inhibitor of nitric oxide synthesis) with the pattern of vastus lateralis deoxygenation
Roberson et al. (2018) [144]	17	Runners (4 F/5 M with protein supplement, 33 ± 7 years, 65.9 ± 11.8 kg; 3 F/5 M placebo, 28 ± 10 years, 72.7 ± 8.0 kg)	5-km treadmill time trial and mitochondrial capacity determination (by a series of blood pressure cuff oscillations and electrical stimulations) before and after 10 weeks of progressive run training with supplementation (placebo pills or whey protein powder)	OxyMon MkIII (TSI, ΔO_2Hb, ΔHHb, ΔtHb)	Medial gastrocnemius (Sf: NA)	Mitochondrial capacity trended toward improving over time with no difference between groups
Rodrigo-Carranza et al. (2021) [145]	10	Well-trained middle- and long-distance runners (5 F/ 5 M: 21.2 ± 1.3 years, 56.6 ± 9.1 kg)	Incremental VO_2max treadmill running test (slope 1%; speed increase 0.28 m/s every min until exhaustion	Humon Hex[d] (SmO_2)	Vastus lateralis (Sf: NA)	Determination of SmO_2 breakpoint by muscle oximetry comparable with 2nd GET

Rowland et al. (2023) [146]	9	Physically active subjects (M: 21 ± 1 years, 77.4 ± 12.5 kg)	After 7 days of supplementation with dietary nitrate-rich or dietary nitrate depleted beetroot powder, 2 h moderate-intensity cycling, followed by a 60 s maximal-intensity end-sprint, with supplements ingested 2 h before and 1 h into the moderate-intensity exercise bout	PortaMon[d] (TSI, ΔO_2Hb, ΔHHb, ΔtHb)	Right vastus lateralis (Sf: NA)	No muscle oxygenation differences during moderate-intensity cycling, but faster HHb kinetics during initial stages of the 60 s end-sprint in nitrate supplementation subjects
Salzmann et al. (2021) [147]	12	Trained runners (M: 24.3 ± 2.7 years, 74.9 ± 8 kg)	Two bouts of 10 min heavy intensity ramp cycle ergometer exercise (60 W with a 1 W increment every 2 s until volitional termination) without mBF restriction, with 40% or 50% mBF restriction	PortaLite (TSI, ΔO_2Hb, ΔHHb, ΔtHb)	Right vastus lateralis muscle (Sf: NA)	mBF restriction applied during cycling exercise in the heavy intensity domain shifted the working muscles to an O_2 dependent situation as evidenced by a greater O_2 extraction
Solsona et al. (2021) [148]	13	Moderately trained runners (M: 24 ± 3 years, 73.8 ± 6.5 kg)	Five bouts of 30 s all-out exercises with lower limb bilateral 60 % mBF restriction during the 1st 2 min of recovery, with gravity-induced mBF restriction (pedaling in supine position), in hypoxia (FiO_2≈13%) or normoxia	OxiplexTS (SO_2, atHb, aO_2Hb, aHHb)	Vastus lateralis (Sf: NA)	aO_2Hb increased in the last two sprints. aO_2Hb availability during sprints decreased during hypoxia. Lower SO_2 with gravity-induced mBF restriction
Solsona et al. (2022) [149]	13	Moderately trained runners (M: 24 ± 3 years, 73.8 ± 6.5 kg)	Five all-out 30-s efforts interspaced with 4 min of passive recovery under four modalities: 60% mBF restriction, gravity-induced mBF restriction, hypoxia (FiO_2 ~ 13%), normoxia.	OxiplexTS (SO_2, atHb, aO_2Hb, aHHb)	Vastus lateralis (Sf: NA)	Greater deoxygenation rates in normoxia compared to mBF restriction. Deoxygenation rates not different between the conditions when adjusted with sprint total work. mBF restriction conditions impaired reoxygenation
Sweeting et al. (2017) [150]	7	Team-sport plus endurance athletes (1 F/6 M: 27.0 ± 6.6 years, 75.1 ± 10.2 kg)	Repeated and single sprint efforts, embedded in a simulated team-sport running protocol, on a non-motorized treadmill in normoxia and hypoxia (simulated altitudes of 2,000 and 3,000 m)	OxyMon MkIII (maximal ΔHHb after pressure cuff inflation post-exercise)	Vastus lateralis (Sf: 5.7 ± 1.4 mm)	Maximal ΔHHb lowered at 3000 m compared to 2000 m and sea-level, as well as for the mean total work performed during the protocol
Woorons et al. (2021) [151]	8	Moderately to well-trained runners (M: 27.1 ± 9 years, 67.3 ± 5 kg)	Two exercises of 10-12 running bouts at either 60, 80 and 100% of maximal aerobic velocity	PortaMon (ΔHHb, ΔtHb)	Right vastus lateralis (Sf: NA)	HHb and tHb changes respectively higher and lower with voluntary hypoventilation

				performed in three sessions 48-72 h apart: 1st exercise performed with so-called voluntary hypoventilation at low lung volume, the 2nd in normal breathing			
	Yáñez-Sepúlveda et al. (2022) [152]	14	Trained subjects (M: 8 trained, 27.7 ± 2.2 years, 75.6 ± 6.7 kg; 6 controls, 25.8 ± 2.6 years, 74.3 ± 5.9 kg)	1.5-mile running test on 400 m Olympic track after 4 weeks concurrent training (five 80-min sessions/week), and inspiratory muscle training (resistance load at 50% and 15% of the maximum dynamic inspiratory strength) for trained and control, respectively	PortaMon (TSI and ΔHHb during ischemia and reperfusion in a venous occlusion test)	Vastus lateralis (Sf: 9.1 ± 2.6 experimental, 10.5 ± 1.9 mm control)	TSI changes moderately correlated with running time
	Zinner et al. (2017) [153]	13	Moderately-to-well-trained team-sport athletes (6 F/ 7 M: 24 ± 2 years, 67.9 ± 8.1 kg)	16 × 30 m all-out sprints (15 s rest) with multidirectional changes of direction movements on a speed court with ischemic preconditioning (3 × 5 min) of legs or of arms 45 min before sprints and control trial	Moxy[d] (SmO_2)	Vastus lateralis, biceps brachii (Sf: NA)	No vastus lateralis and biceps brachii SmO_2 differences between the three conditions as for sprint performance
**Speed skating**	Rębis et al. (2022) [154]	27	National teams speed skaters (13 F: 18.5 ± 2.0 years, 60.4 ± 3.6 kg; 14 M: 17.9 ± 2.2 years, 73.9 ± 6.5 kg)	Wingate test and graded exercise test to exhaustion performed on a cycle ergometer	Moxy[d] (SmO_2)	Vastus lateralis (Sf: NA)	SmO_2 not different between tests. Gender not differentiating factor in SmO_2 changes regardless of test type. Negative correlation between SmO_2 half time required to reach maximal value with VO_2max and test duration
**Sprint (track field)**	Yamaguchi et al. (2021) [155]	9	Trained 100-200 m sprinters (M: 19.3 ± 0.4 years, 63.8 ± 2.2 kg)	Three 15-s maximal cycling sprints interspersed with 7-min recovery in control (23°C, FiO_2 20.9%), hypoxic (23°C, FiO_2 14.5%), and hot hypoxic (35°C, FiO_2 14.5%) environments	Hb14 (StO_2)	Right vastus lateralis (Sf < 7 mm)	StO_2 reduction greater in hot/hypoxic than in hypoxic conditions during the 2nd sprint

**Sprint canoe-kayak**	Paquette et al. (2018) [156]	30	Highly trained sprint canoe-kayak athletes (19 kayakers and 11 canoeists, 21 ± 3 years, 74.6 ± 10.9 kg (M: kayakers 79.9 ± 6.9 kg, M: canoeist, 78.9 ± 15.1 kg, F: kayakers 68.2 ± 8.8 kg, F: canoeist 66.0 ± 9.2 kg)	200- and 500- (for women), and 200- and 1000-m (for men) on-water time trial	Moxy[d] (SmO_2)	Latissimus dorsi, biceps brachii, vastus lateralis (Sf < 25 mm). Dominant side (kayakers) and front leg, opposite biceps brachii and latissimus dorsi for canoeists	Lower SmO_2 for 3 muscles in kayakers compared to canoeists. Muscle maximal O_2 extraction, a better predicator of performance than VO_2max in sprint canoe-kayak
	Paquette et al. (2019) [157]	13	Well-trained canoe-kayak athletes (9 kayakers (3 F/6 M) and 4 canoeists (2 F/2 M): 22 ± 3 years, 71.5 ± 8.3 kg)	4 high-intensity interval training sessions and sprint interval training on a canoe or kayak ergometer	Moxy[d] (SmO_2)	Kayakers: latissimus dorsi, biceps brachii, vastus lateralis; canoeists: dominant side front leg, opposite biceps brachii, latissimus dorsi (Sf < 25 mm)	Short interval sessions elicited both lowest deoxygenation and longest time spent at low SmO_2 levels
	Paquette et al. (2020) [158]	8	Elite middle and long-distance runners (M: 26.3 ± 5.1 years, 69.2 ± 8.6 kg)	200-m and 1,000-m on-water time trial before and after a 3-weeks winter training camp	Moxy[d] (SmO_2)	Biceps brachii (Sf < 25 mm)	Higher vastus lateralis SmO_2 during exercise in group with nitrate-rich beetroot supplementation
**Swimming**	Dalamitros et al. (2021) [159]	12	National-level swimmers (9 M: 21.9 ± 2.0 years, 78.8 ± 9.8 kg)	Submaximal (8 efforts of 50 m) or a maximal interval (4 efforts of 15 m), followed by two series of 4 maximal 25 m efforts	Moxy[d] (SmO_2)	Dominant arm deltoid (Sf: NA)	SmO_2 correlated with blood lactate and heart rate values after maximal protocol
	Grossman et al. (2021) [160]	10	Trained subjects (M: 24 ± 3 years)	4-6 min trials of heavy-intensity cycling with: (1) continuous free breathing; (2) continuous with 5 s breath-hold every 25 s; (3) Fartlek, a 5 s sprint followed by 25 s of heavy-intensity cycling; 4) a combined Fartlek and breath-hold	OxiplexTS (SO_2, atHb, aO2Hb, aHHb)	Vastus lateralis (Sf: NA)	SO_2 and tHb lower in continuous free breathing with breath-hold compared with continuous free breathing, whereas aHHb greater in Fartlek with breath-hold compared with Fartlek
	Jones et al. (2018) [161]	14	Junior club level swimmers (5 F/9 M: 15.3 ± 1.7 years, 64.2 ± 11.0 kg)	5 X 100 m maximal freestyle exercise interspersed with 3 min recovery periods before and after an eight-week high volume endurance training period (35 ± 8 km/ week)	PortaMon[d] (TSI, ΔO_2Hb, ΔHHb, ΔtHb)	Dominant leg vastus lateralis (M Sf: 13.6 ± 4.4 mm; F Sf: 18.8 ± 5.1 mm)	Increased in HHb during individual sprints. Faster TSI recovery time. Correlation ($r = 0.72$) between increased reoxygenation rate and improved swim performance time

	McGowan et al. (2017) [162]	25	Elite swimmers (13 F: 20 ± 2 years, 12 M: 20 ± 3 years)	30-min transition phase wearing tracksuit jacket with heating elements and performing dry land-based exercise routine, or conventional tracksuit and remaining seated; 100-m freestyle time trial	PortaMon[d] (ΔHHb, ΔtHb, ΔHbdiff)	Trapezius (Sf: NA)	tHb increase combining traditional pool warm up with passive heating via heated jackets and completion of dry land-based exercises; improvement in performance by 0.8%
	Pratama et al. (2020) [163]	12	Collegiate swimmers (6 F/6 M: 18.5 ± 0.6 years, 62.0 ± 6.5 kg)	3 sessions of 2 consecutive 200-m front-crawl trials separated by different recovery protocols	PortaMon[d] (ΔTSI, ΔO_2Hb, ΔHHb, ΔtHb)	Biceps femoris (Sf: 14.7 ± 0.7 mm)	O_2Hb and TSI recovered or progressively increased after 200-m swim trial regardless of recovery interventions, with a greater muscle reoxygenation rate during active recovery and combination of active/passive recovery when compared with passive recovery
**Team sports**	Chang et al. (2020) [164]	47	23 Taiwan national basketball, badminton and football teams' athletes, 24 moderately active (23 F, 19 ± 3 years, 24 F, 24 ± 2 years)	High-intensity interval treadmill testing (alternation between 1-min effort at 120% of maximal speed, and 1-min rest) until volitional exhaustion	PortaMon[d] (TSI, ΔO_2Hb, ΔHHb, ΔtHb)	Right vastus lateralis (Sf: NA)	Variance in number of repetitions completed within the cohort was related to TSI at exhaustion. TSI at exhaustion was one of the major factors related to high-intensity interval treadmill testing exercise (r = 0.45)
	Cyr-Kirk et al. (2022) [165]	13	Badminton, basketball, and hockey team sport athletes (5 F/8 M: 23.0 ± 2.7 year, 70.7 ± 14.9 kg)	Five 6-s all-out sprints with 24-s recovery until exhaustion under normoxic and hyperoxic (FiO_2 40%) conditions	PortaMon[d] (TSI, ΔO_2Hb, ΔHHb, ΔtHb)	Vastus lateralis (Sf: 9.0 ± 4.6 mm)	Hyperoxic condition increased the amplitude of TSI, HHb changes during sprints and recovery periods
	Dennis et al. (2023) [166]	13	Amateur team-sport players (13 M: 20.2 ± 3.0 years, 82.2 ± 7.8 kg)	3 × 5 × 10-s maximal cycle sprints, with 20-s passive recovery between sprints, and 5 min active recovery between sets at simulated altitude of ~3000 m and air temperatures of 20, 35 and 40°C	Moxy[d] (SmO_2)	Right vastus lateralis (Sf: NA)	Muscle deoxygenation during exercise, and reoxygenation during recovery of greater magnitude in 35° and 40° than 20°C
	Fryer et al. (2019) [167]	9	Amateur level team sport athletes (M: 20.7 ±1.1 years, 84.0 ± 14.2 kg)	4-weeks 30-min handgrip training 4 times/week in hypoxic (14% FiO_2) and normoxic conditions. mBF, mVO_2 and perfusion assessed pre, post 4-weeks training, and following 4-week detraining	PortaLite (ΔO_2Hb, ΔHHb, ΔtHb, mBF, mVO_2)	Flexor digitorum profundus (Sf: 4.2 ± 0.9 mm)	mVO_2 and perfusion increased at faster rate with hypoxic training. Increased mBF for hypoxic condition. During 4-weeks detraining, mVO_2 and perfusion declined at similar rates for both conditions, whereas mBF decreased significantly faster following hypoxic training. Four weeks hypoxic

							training increased peripheral O_2 delivery and utilisation
	Gatterer et al. (2018) [168]	11	Amateur level basketball, football and handball athletes (M: 24.0 ± 2.4 years, 84.0 ± 9.3 kg)	Training:<br>Repeated-sprint or sprint interval training in hypoxia cycling training for 3 weeks at a simulated altitude of 2200 m. Before and 3 days after training, Wingate and repeated cycling sprint test (5 x 6 s, 20 s recovery). Five days after training, repeated running sprint test (6 x 17 m back and forth, 20 s recovery) and Yo-Yo intermittent recovery test	NIRO-200 (TOI, ΔtHb)	Vastus lateralis (Sf: NA)	Repeated-sprint and sprint interval training result in different patterns of muscular O_2 extraction. Repeated sprint compared to sprint interval training in hypoxia induced greater deoxygenation and re-oxygenation during repeated cycling sprints. Improvements in cycling performance were associated with re-oxygenation during the sprints after training ($r > 0.7$)
	Jeffries et al. (2019) [169]	20	Trained team sports university athletes (M: 10 treated, 22 ± 3 years, 80.7 ± 10.3 kg; M: 10 sham, 21 ± 2 years, 87.5 ± 20.6 kg)	Prior to and 72-h following the intervention, participants performed submaximal cycling at 70, 80 and 90% of GET followed by an incremental exercise test.<br>Intervention: 7-d repeated bilateral lower limb occlusion (4 x 5-min) of ischemic preconditioning (n: 10) or sham (n: 10)	PortaMon[d] (ΔO_2Hb, ΔHHb, ΔtHb, mBF, mVO_2)	Vastus lateralis (Sf: NA)	HHb decreased following ischemic preconditioning ~ 30% with no tHb changes
**Triathlon**	Contreras-Briceño et al. (2022) [170]	15	Competitive triathletes (7 F/8 M: 29 ± 6 years, 69.2 ± 9.4 kg)	Ramp incremental test on cycle ergometer to exhaustion (20 W/80 s)	Moxy[d] (SmO_2)	Intercostal of right 7th intercostal space (Sf: NA)	SmO_2 decreased continuously and followed lung ventilation changes (determination of respiratory compensation point)
	Olcina et al. (2019) [171]	10	Trained Sprint/Olympic distance triathletes (2 F/8 M: 25.7 ± 8.9 years, 71.3 ± 9.8 kg)	Isolated 12-min maximal running on 400 m track, and bike-run trial separated by 7 days. Bike: 20-min time trailer on a trainer	Moxy[d] (SmO_2)	Vastus lateralis (Sf: NA)	Lower SmO_2 during isolated run than during bike-running trial
	Paradis-Deschênes et al. (2020) [172]	27	Trained cyclists, runners, and swimmers (M: 18 athletes, 9 controls; age and weight NA)	8 training sessions in 4 weeks. Sprint interval training preceded by bilateral ischemic preconditioning (3 x 5 min ischemia/5 min reperfusion cycles). Pre-, mid, and post-training 30 s	PortaMon[d] (TSI, ΔHHb, ΔtHb)	Right vastus lateralis (Sf: 5.3 ± 0.7 mm athletes, 6.7 ± 0.6 controls)	4 weeks of bilateral ischemic preconditioning applied before sprint interval training enhanced local perfusion, quadriceps O_2 extraction and elicited greater gains in time-trial performance

				Wingate test, 5-km time trial, and maximal incremental cycling step test (30 W/min)			
	Paradis-Deschênes et al. (2020) [173]	9	Trained road cyclists, runners, and triathletes (M: 26.4 ± 1.6 years, 75.5 ± 3.5 kg)	Two 5-km cycling time trials interspersed by 45 min recovery including ischemic preconditioning, active recovery or neuromuscular electrical stimulation	PortaMon[d] (TSI, ΔO_2Hb, ΔHHb, ΔtHb)	Right vastus lateralis (Sf: 6.4 ± 0.6 mm)	Enhanced tHb following 3 recovery modalities; no difference in TSI and muscle O_2 extraction during exercise
	Triska et al. (2021) [174]	9	Endurance-trained triathletes (M: 27.7 ± 4.3 years, 76.5 ± 5.6 kg)	30-min single-visit protocol (time trials of 10, 5 and 2 min interspersed with 30 min rest); multivisit protocol (time trials of 10, 5 and 2 min in randomized order interspersed by > 24 h rest)	PortaMon[d] (ΔHHb)	Right vastus lateralis (Sf: NA)	HHb changes were not different between protocols
**Ultra running**	Giovannelli at al. (2020) [175]	18	Experienced ultra-endurance runners (1 F/17 M: 36.8 ± 9.2 years, 68.1 ± 8.1 kg)	Incremental uphill treadmill running test before trail running competition, and incremental exercise until exhaustion by utilizing a one-leg knee-extension ergometer (workload increase by ~8 W/min) before and after trail running competition of 32- or 50-km with 2000 m or 3500 m of elevation gain, respectively	PortaLite (ΔHHb, mVO_2)	Vastus lateralis (Sf: NA)	After competition, ΔHHb increase as a function of work rate, from unloaded to peak, is less pronounced. Lower peak ΔHHb values lower after competition. No significant differences in peak ΔHHb between subjects participating in 30 or 50 km race
	Kerhervé et al. (2017) [176]	8	Trained ultramarathon runners (3 F/5 M: 37.4 ± 7.4 years, 70.5 ± 8.9 kg)	6 hours treadmill run at moderate and heavy exercise intensities	PortaMon[d] (TSI)	Vastus lateralis, gastrocnemius (Sf: 4.1 ± 1.2, 4.8 ± 3.1 mm)	No effect of exercise duration on TSI
	Vernillo et al. (2017) [177]	26	Runners (14 M: 47.6 ± 7.2 years, 68.4 ± 6.9 kg); controls (12 M: 32.7 ± 7.1 years, 73.2 ± 8.0 kg)	Two 4-min constant-load cycling bouts at power outputs of 1 and 1.5 W/kg before and after 330 km extreme ultratrail running	NIMO (SmO_2, atHb, aO_2Hb, aHHb)	Dominant leg vastus lateralis (Sf: 6.3 ± 2.5 runners, 6.9 ± 2.4 mm controls)	After extreme running, SmO_2 decreased (increase in aHHb) only in ultratrail runners
**Cross-country running and swimming/**	Meyer et al. (2021) [178]	19	Cross-country runners (6 M: 19.3 ± 0.8 years, 57.3 ± 6.1 kg), upper-body	Short bout of exercise followed by 15-20 (5-10 s) arterial occlusions. Gastrocnemius exercised	OxyMon MkIII (ΔO_2Hb, ΔHHb)	Right medial gastrocnemius, vastus lateralis, biceps	Higher muscle oxidative capacity of lower limb muscles when compared to upper limb muscles regardless of

**rowing**			endurance training group swimming/rowing (5 M: 26.0 ± 6.3 years, 79.0 ± 11.0 kg), controls (8 M: 22.8 ± 2.1 years, 79.4 ± 8.2 kg)	by repeated plantar flexion, vastus lateralis by full leg extension, biceps brachii by arm flexion, and wrist flexor by wrist flexion		brachii, flexor carpi radialis (Sf_u: 4.6 ± 0.7, 4.2 ± 0.7, 3.4 ± 0.3, 3.0 ± 0.5 mm)	specific training. Higher oxidative capacities in lower limb or upper limb muscles of athletes when compared to controls
**Cycling and running**	Feldman et al. (2022) [179]	10	5 recreationally trained cyclists: 1 F/4 M: 4 M; 5 not recreationally trained runners: 3 F/2 M; F: 26.5 ± 4.2 years, 55.5 ± 3.9 kg; M: 29.3 ± 6.6 years, 74.8 ± 9.5 kg)	Incremental cycling and treadmill tests	Moxy[d] (SmO_2)	Right and left vastus lateralis (Sf: NA)	NIRS derived breakpoints are suitable to determine GET 1 and 2
**Cycling and triathlon**	Schörkmaier et al. (2021) [180]	13	Experienced cyclists or triathletes (M: 25.2 ± 3.5 years, 71.0 ± 8.0 kg)	4 maximal graded incremental exercise tests on cycle ergometer under normoxic or hypoxic conditions, either with nonivamide-nicoboxil or placebo cream	Moxy[d] (SmO_2)	Bilateral vastus lateralis (Sf_u: < 12 mm)	Application of nonivamide-nicoboxil cream increased SmO_2 at rest and during different submaximal workloads as well as during physical exhaustion, irrespective of normoxic or hypoxic conditions. No increase in endurance performance
**Handball and football**	Tong et al. (2019) [181]	9	Trained handball and football players (M: 20.6 ± 0.9 years, 68.8 ± 8.8 kg)	Simulated team-sport 25-min intermittent exercise in 2 phases, on a non-motorized treadmill, interspersed by a 15-min half-time break. During this interval, players rested passively or performed 4-min core muscle exercise concomitant with inspiratory loaded breathing following 11-min passive recovery.	NIRO-200 (ΔO_2Hb, ΔHHb, ΔtHb)	Left vastus lateralis (Sf: 7.7 ± 3.1 mm)	Vastus lateralis oxygenation following re-warm-up exercises not different from that of passive recovery
**Judo and Cycling**	Anthierens et al. (2019) [182]	30	Highly trained judokas (11 M: 20.3± 0.7 years, 64.2 ± 1.9 kg), competitive cyclists (10 M: 19.7 ± 0.4 years, 78.1 ± 3.6 kg), untrained controls (9 M: 23.1 ± 0.3, 68.4 ± 2.7 kg)	Trunk extension exercises on isokinetic dynamometer	PortaMon[d] (ΔHHb, ΔtHb)	Dominant side trunk extensor muscle at level of 3rd lumbar vertebrae (Sf: NA)	Cyclists presented greater trunk extensor muscle deoxygenation and tHb responses compared to untrained men
**Running and Triathlon**	Pawlak-Chaouch et al. (2019) [183]	11	Intense endurance exercise training and competitive running or triathlon	Supramaximal intensity cycling intermittent test until exhaustion (15-s bouts at 170% of maximal	OxyMon MkIII (ΔO_2Hb, ΔHHb, ΔtHb)	Vastus lateralis (Sf: NA)	No tHb and HHb changes during time-matched work + recovery periods from supramaximal intensity cycling tests

			athletes (M: 21.7 ± 3.7 years, 66.8 ± 8.5 kg)	aerobic power interspersed with 30-s passive recovery periods) following either a 3-day Nitrate-rich beetroot juice supplementation or placebo			following beetroot juice supplementation
**Cross-country, CrossFit, and power lifting**	Green et al. (2023) [184]	24	3 groups of trained M athletes: Strength (Olympic lifters or power lifters; 24.3 ± 3.4 years, 99.7 ± 19.2 kg); Endurance (cross-country runners; 22.9 ± 6.8 years, 68.7 ± 6.8 kg); high-intensity functional training (CrossFit; 30.5 ± 5.7 years, 86.8 ± 10.5 kg)	Rate measurement of mVO_2 recovery back to resting level by a series of 5-10 s arterial occlusions (250–300 mmHg) following contractions induced by 30-s surface neuromuscular electrical stimulation	OxyMon MkIII (ΔO_2Hb, ΔHHb, ΔtHb, mVO_2)	Right vastus lateralis (Sf: NA)	Lower mVO_2 recovery rate to resting levels following surface neuromuscular electrical stimulation in strength compared to endurance group; high-intensity functional training group trending toward a higher mVO_2 recovery rate compared to strength group
**More than three**	Lagerwaard et al. (2019) [185]	16	Low- (sailing, running, weightlifting, volleyball) and relatively high- (cycling, lacrosse, triathlon, rowing, running) aerobic fitness subjects (M: 8 low-fitness, 24.1 ± 2.7 years, 80.0 ± 8.3 kg; 8 high-fitness, 22.6 ± 4.2 years, 73.3 ± 6.4 kg)	Three 30-s rest measurements of basal mVO_2. Minimal oxygenation determined by 30-s maximal hand grip exercise for flexor digitorum or by plantar flexion exercise for gastrocnemius, followed by an arterial occlusion. Recovery O_2 consumption after exercise measured after 30 s of intermittent handgrip exercise at 50% of MVC or plantar flexion exercise until 50% of maximal oxygenation. After exercise, a series of transient occlusions (5 × 5 s on/5 s off, 5 × 7 s on/7 s off, 10 × 10 s on/10 s off) to measure mVO_2 recovery	PortaMon[d] (ΔO_2Hb, ΔHHb, mVO_2)	Gastrocnemius, flexor digitorum superficialis (Sf: low fitness, 15.0 ± 2.2, 7.2 ± 2.8; high fitness, 9.1 ± 4.1, 5.0 ± 1.6 mm)	Mitochondrial capacity higher in high-fitness individuals compared to low-fitness individuals in the gastrocnemius (correlated with VO_2peak), but not in flexor digitorum superficialis
	Lagerwaard et al. (2021) [186]	32	Low- (sailing, running, weightlifting, volleyball) and relatively high- (cycling, lacrosse, triathlon, rowing, running) aerobic fitness subjects (16 low-fitness, 24.0 years, 59.2 ± 7.2 kg; 16 high-fitness,	mVO_2 calculated during arterial occlusion using slope of the change in HHb and O_2Hb for 3 s for the 5 s occlusions, for 5 s for the 7 s occlusions, 7 s for the 10 s occlusions and 15 s for the basal measurements	OxyMon MkIII (TSI, ΔO_2Hb, ΔHHb, ΔtHb, mVO_2)	Medial gastrocnemius, wrist flexors (Sf: gastrocnemius, 8.6 and 6.9 mm; wrist flexors, 5.3 and 4.0 mm)	mVO_2 recovery faster in high- compared to low-fitness group in gastrocnemius, but not in wrist flexors. VO_{2peak} correlated to mVO_2 recovery in both muscles

		21.8 years, 60.8 ± 6.9 kg, all F)				
Rodriguez et al. (2019) [187]	10	Combat sports, CrossFit, road cycling, and team sports trained athletes (M: 25.5 ± 3.6 years, 81.45 ± 8.29 kg)	3 sessions of ten 10-s sprints on a cycle ergometer: 1st and 2nd sessions "all-out' efforts without or with inspiratory loading, 3rd session ten 10-s work-matched intervals	OxyMon MkIII (TSI, ΔHHb)	Vastus lateralis (dominant leg), intercostal of 6th left intercostal space (Sf: 11.9 ± 6.9, 11.2 ± 4.4 mm)	During work-matched intervals exercise, no differences in vastus lateralis TSI to exercise with inspiratory loading for both sprint and recovery phases. Higher intercostal TSI during work-matched intervals exercise compared to exercise with inspiratory loading, and lower HHb. No difference in reoxygenation phase between the two conditions of inspiratory loading
Rodriguez et al. (2019) [188]	10	Road cycling, strength training and team sports (M: 10, 26.6 ± 3.6 years, 78.6 ± 9.4 kg)	Ten 10-s sprints separated by 30-s rest in normoxia or hypoxia (FiO_2 15%)	OxyMon MkIII (ΔO_2Hb, ΔHHb, ΔtHb)	Vastus lateralis (dominant leg), intercostal of 6th intercostal space (Sf: 12 ± 7, 11 ± 4 mm)	Hypoxia did not affect intercostal muscle oxygenation, and decreased vastus lateralis oxygenation during repeated-sprint exercise
Ruiz-Moreno et al. (2022) [189]	15	Cycling, team sports and triathlon moderately trained subjects (7 F/8 M: 31 ± 6 years, 67.4 ± 12.3 kg)	Cycling, team sports and triathlon moderately trained subjects (7 F/8 M: 31 ± 6 years, 67.4 ± 12.3 kg)	Moxy[d] (SmO_2)	Bilateral vastus lateralis (Sf: left = 5.6 ± 1.8 mm; right = 5.6 ± 2.3 mm)	SmO_2 changes unaffected by caffeine; caffeine increased post-exercise blood lactate concentration and peak and mean cycling power during a 15-s Wingate test
Trexler et al. (2020) [190]	27	Resistance, endurance exercise and recreational sports active subjects (M: 22 ± 4 years, 78.9 ± 12.5 kg)	Two hours after supplementation with citrulline malate or beetroot juice, 3-min submaximal isotonic leg extensions (every 4 s from 90 to 150° angle) at 25% of MVC	PortaLite (ΔO_2Hb, ΔHHb, ΔtHb, mBF, mVO_2)	Right vastus lateralis (Sf: 5 ± 2 mm)	Baseline mBF and mVO_2 resting values did not differ among treatments. No treatment effect for mBF and mVO_2 during submaximal exercise
Willis et al. (2019) [191]	7	Climbing, cycling, cross-country skiing, resistance-training running and swimming physically active subjects (M: 26.6 ± 2.9 years, 74.0 ± 13.1 kg)	Four sessions of repeated sprint tests (10-s maximal sprints with 20-s recovery until exhaustion) during both leg and arm cycling with bilateral mBF restriction (occlusion at 0% vs. 45% of resting pulse elimination pressure) and systemic hypoxia (FiO_2 13.1 %) or normoxia	PortaMon[d] (TSI, ΔO_2Hb, ΔHHb, ΔtHb)	Vastus lateralis, biceps brachii (Sf: NA)	Biceps brachii HHb, tHb and TSI changes greater than vastus lateralis ones. Greater tHb changes during mBF restriction only compared to all other conditions
Willis et al. (2019) [192]	16	Climbing, cycling, cross-country skiing, resistance-training, running, and swimming trained	Repeated sprint arm cycling test to exhaustion (10 s maximal sprints with 20 s recovery until exhaustion) in 4 conditions with	PortaMon[d] (TSI, ΔO_2Hb, ΔHHb, ΔtHb)	Biceps brachii (Sf: NA)	Increased changes in blood perfusion (tHb) between minimum-maximum of sprints with flow restriction (400 m, 45% and 3800 m, 45%) than without

			subjects (5 F/11 M: 26.4 ± 4.0 years, 73.8 ± 9.8 kg)	2 levels of normobaric hypoxia (400 and 3800 m) and 2 levels of mBF restriction (0% and 45% of total occlusion)			(400 m, 0% and 3800 m, 0%). ΔTSI decrease more with both mBF restriction conditions than without. Maximum TSI progressively reduced with both mBF restrictions and systemic hypoxia
**Not reported**	Brodeur et al. (2022) [193]	23	Physically active university students (M: 18-24 years)	135 s quadriceps static or dynamic stretching. Static stretch: 3 times 45-s stretch with 30-s standing rest; dynamic stretch: walking by flexing knee then grabbing ankle before quickly releasing the stretch repeated over the course of 10 yards and back for entire period with no rest between stretch	Moxy[d] (SmO_2)	Rectus femoris (Sf: NA)	Higher SmO_2 increase post-stretch after dynamic stretching as compared to static stretching. SmO_2 increase after dynamic stretching persisted for 2 min
	Kojima et al. (2020) [194]	25	Healthy and active subjects with no formal physical training (M: 24 ± 2 years, 66.1 ± 7.5 kg)	Repetitions of the one-arm preacher curl at 30% of their 1-repetition maximum until exhaustion under hyperoxic (FiO_2 30%) and normoxic conditions	NIRO-200NX (ΔO_2Hb, ΔHHb, ΔtHb)	Right biceps brachii (Sf: NA)	No blood O_2 content effect in any NIRS parameters. Time effect in all NIRS parameters. Interaction effect in O_2Hb and tHb, but not in HHb. No difference between hyperoxia and normoxia values at any measurement points for ΔtHb, but the hyperoxia value in O_2Hb lower than the normoxia value at the maximum number of repetitions under normoxia
	Lagerwaard et al. (2020) [195]	40	Low to moderate physical active young and old subjects (20 M: 22 ± 2.0 years; 20 M: 69 ± 1.9 years)	mVO_2 calculated during arterial occlusion using the slope of the change in HHb and O_2Hb for 3 s for the 5 s occlusions, for 5 s for the 7 s occlusions, 7 s for the 10 s occlusions and 15 s for the basal measurements	PortaMon[d] (ΔO_2Hb, ΔHHb, mVO_2)	Vastus lateralis, gastrocnemius, tibialis anterior (Sf: Young, 10.4 ± 2.7, 10.4 ± 2.8, 9.3 ± 3.1; Old, 9.1 ± 2.7, 7.6 ± 2.4, 8.5 ± 3.7 mm)	Mitochondrial capacity lower in older group in gastrocnemius and vastus lateralis, but not in tibialis anterior. Reperfusion rate not different for gastrocnemius, but faster in tibialis anterior and vastus lateralis in young group compared to older group
	Le Roux-Mallouf et al. (2020) [196]	24	Moderately trained subjects (12 F: 25 ± 1 years, 59.1 ± 6.2 kg; 12 M: 26 ± 3 years, 76.4 ± 5.1 kg)	Quadriceps 35% MVC isometric unilateral knee extension (5-s contraction/4-s relaxation, increase by 5% every 4 min), and incremental cycling exercise tests to exhaustion before and after a 2-month exercise training program and daily intake of	OxyMon MkIII (TSI, ΔO_2Hb, ΔHHb, ΔtHb)	Right vastus lateralis (Sf: NA)	Nitrate and citrulline supplementation had no effect on muscle oxygenation during both exercises

			a placebo or a nitrate-rich salad and citrulline drink			
Lin et al. (2023) [197]	35	Physically active young (17) and old (18) subjects (10 F/7 M young: 19.8 ± 1.6 years, 62.5 ± 10.5 kg; 11 F/7 M older: 61.5 ± 5.1 years, 63.8 ± 9.6 kg)	Isometric knee extension tests involving voluntary contraction intensity (20% MVC for 30 s) and 30-s electrical stimulation before and after multicomponent exercise training for 12 weeks (2-3 sessions/week)	Imagent (SO_2, ΔO_2Hb, ΔHHb, ΔtHb)	Vastus lateralis (Sf: young, 6.7 ± 2.5; old, 7.8 ± 3.6 mm)	Young adults post-training SO_2 upon electrical stimulation increased and higher in older adults. Post-training recovery phase inflection time of voluntary contraction shorter than that of pre-training period
Morishima et al. (2020) [198]	19	Physically active subjects (M: 10 fish oil group, 20.4 ± 0.7 years, 68.5 ± 10.3 kg; 9 placebo, 21.2 ± 2.0 years, 65.3 ± 10.9 kg)	Repeated knee extensions with weights equal to 40% of body weight 8 weeks after eicosapentaenoic acid and docosahexaenoic acid, or placebo supplementation	Hb11 (StO_2)	Left vastus lateralis (Sf: NA)	No StO_2 difference during muscular endurance test between groups
Mota et al. (2020) [199]	20	Physically active subjects (F: 23.1 ± 3.3 years, 63.3 ± 9.1 kg)	3-min maximal effort arm cycling exercise, preceded by ischemic preconditioning on both arms or sham	PortaMon[d] (ΔO_2Hb, ΔHHb)	Right triceps brachialis (Sf_u < 10.8 mm)	No O_2Hb and HHb time course differences between conditions, as well as in performance indicators
Nell et al. (2020) [200]	20	Healthy subjects (9 F/11 M: 25 ± 3 years, 70.1 ± 12.0 kg)	Handgrip exercise at 50% of MVC (duty cycle: 2 s:1 s) until task failure during hypoxia (FiO_2 12%) or normoxia	PortaLite (TSI, ΔO_2Hb, ΔHHb, ΔtHb)	Flexor digitorum superficialis (Sf_u: 3.1 ± 1.1 mm)	Hypoxic compared to normoxic handgrip exercise induced a lower flexor digitorum TSI and both protocols decreased flexor digitorum TSI from baseline to task failure. Under both hypoxia and normoxia, HHb and tHb increased from baseline to task failure whereas O_2Hb decreased and then increased during handgrip exercise
Papadopoulos et al. (2018) [201]	16	Recreationally active subjects performing regular aerobic exercise at moderate intensity (M: 21.9 ± 0.5 years, 75.9 ± 4.8 kg)	After 2.5-hours nitrate-rich supplementation or control, 3-min sustained isometric handgrip exercise (30% MVC) followed by sustained isometric handgrip exercise (with arterial occlusion) to exhaustion	OxyMon MkIII (ΔO_2Hb, Δ HHb, ΔtHb, mVO_2)	Flexor digitorum profundus (Sf: 4.3 ± 0.8 mm)	Beetroot juice improved muscle performance and oxygenation during sustained isometric exercise, but did not alter muscle oxidative efficiency (magnitude and slope of O_2Hb)

a absolute, *CV* coefficient of variation, *Δ* relative changes, *GET* gas exchange threshold, *HHb* deoxyhemoglobin, *O_2Hb* oxyhemoglobin, *tHb* total hemoglobin = O_2Hb+HHb, Hbdiff = O_2Hb-HHb, *F* female, *M* male, *mBF* muscle blood flow, *mVO_2* muscle oxygen uptake, *MVC* maximal voluntary contraction, *NA* subcutaneous fat tissue measurement not available, *NIRS* near-infrared spectroscopy, *O_2* oxygen, *rSO_2/SmO_2/StO_2/SO_2TOI/TSI* muscle oxygen saturation %, *rpm* revolutions per minute, rpm revolutions per min, *SD* standard deviation,

Sf subcutaneous fat tissue depth measured by Harpenden calipometry through skinfold thickness from under the optode on the muscle, *Sfu* subcutaneous fat tissue depth measured by Doppler ultrasound from under the optode on the muscle, VO_2max maximal oxygen uptake, *W* Watt

[a]Some studies reported muscle oxygenation changes before and after either a training or treatment (e.g. supplementation) period. Oxygenated and deoxygenated species discrimination between the oxygenated and deoxygenated forms of Hb and Mb, respectively, cannot be made. Therefore, the tissue oxygenation changes reported will contain contributions from both chromophores.

[b]Data are expressed as mean ± SD

[c]See also Table 2

[d]Wearable NIRS system with wireless data transmission
